# A *Pex7* Deficient Mouse Series Correlates Biochemical and Neurobehavioral Markers to Genotype Severity—Implications for the Disease Spectrum of Rhizomelic Chondrodysplasia Punctata Type 1

**DOI:** 10.3389/fcell.2022.886316

**Published:** 2022-07-11

**Authors:** Wedad Fallatah, Wei Cui, Erminia Di Pietro, Grace T. Carter, Brittany Pounder, Fabian Dorninger, Christian Pifl, Ann B. Moser, Johannes Berger, Nancy E. Braverman

**Affiliations:** ^1^ Department of Human Genetics, McGill University, Montreal, QC, Canada; ^2^ Department of Medical Genetics, King Abdul-Aziz University, Jeddah, Saudi Arabia; ^3^ Child Health and Human Development Program, Peroxisome Disease Laboratory, Research Institute of the McGill University Health Centre, Montreal, QC, Canada; ^4^ Department of Pathobiology of the Nervous System, Center for Brain Research, Medical University of Vienna, Vienna, Austria; ^5^ Department of Molecular Neurosciences, Center for Brain Research, Medical University of Vienna, Vienna, Austria; ^6^ Hugo W Moser Research Institute, Kennedy Krieger Institute, Baltimore, MD, United States

**Keywords:** rhizomelic chondrodysplasia punctata (RCDP), peroxisome biogenesis disorders, PEX7 gene, adult Refsum’s disease, neurobehavioral phenotypes, plasmalogens, phytanic acid, very long chain fatty acid (VLCFA)

## Abstract

Rhizomelic chondrodysplasia punctata type 1 (RCDP1) is a peroxisome biogenesis disorder caused by defects in *PEX7* leading to impairment in plasmalogen (Pls) biosynthesis and phytanic acid (PA) oxidation. Pls deficiency is the main pathogenic factor that determines the severity of RCDP. Severe (classic) RCDP patients have negligible Pls levels, congenital cataracts, skeletal dysplasia, growth and neurodevelopmental deficits, and cerebral hypomyelination and cerebellar atrophy on brain MRI. Individuals with milder or nonclassic RCDP have higher Pls levels, better growth and cognitive outcomes. To better understand the pathophysiology of RCDP disorders, we generated an allelic series of *Pex7* mice either homozygous for the hypomorphic allele, compound heterozygous for the hypomorphic and null alleles or homozygous for the null allele. Pex7 transcript and protein were almost undetectable in the hypomorphic model, and negligible in the compound heterozygous and null mice. *Pex7* deficient mice showed a graded reduction in Pls and increases in C26:0-LPC and PA in plasma and brain according to genotype. Neuropathological evaluation showed significant loss of cerebellar Purkinje cells over time and a decrease in brain myelin basic protein (MBP) content in *Pex7* deficient models, with more severe effects correlating with *Pex7* genotype. All *Pex7* deficient mice exhibited a hyperactive behavior in the open field environment. Brain neurotransmitters analysis of *Pex7* deficient mice showed a significant reduction in levels of dopamine, norepinephrine, serotonin and GABA. Also, a significant correlation was found between brain neurotransmitter levels, the hyperactivity phenotype, Pls level and the severity of *Pex7* genotype. In conclusion, our study showed evidence of a genotype-phenotype correlation between the severity of *Pex7* deficiency and several clinical and neurobiochemical phenotypes in RCDP1 mouse models. We propose that PA accumulation may underlie the cerebellar atrophy seen in older RCDP1 patients, as even relatively low tissue levels were strongly associated with Purkinje cells loss over time in the murine models. Also, our data demonstrate the interrelation between Pls, brain neurotransmitter deficiencies and the neurobehavioral phenotype, which could be further used as a valuable clinical endpoint for therapeutic interventions. Finally, these models show that incremental increases in *Pex7* levels result in dramatic improvements in phenotype.

## 1 Introduction

Rhizomelic chondrodysplasia punctata type 1 (RCDP1) is a distinct peroxisome biogenesis disorder caused by defects in *PEX7* that impair the peroxisomal pathways of plasmalogen (Pls) synthesis and phytanic acid (PA) oxidation. Clinically, there is a spectrum of phenotype severity that can be distinguished by the degree of Pls deficiency. Although the majority of patients have classic (severe) RCDP, there are patients with non-classic (mild) RCDP.

Classic (severe) RCDP is characterized by congenital cataracts, proximal limb shortening (rhizomelia) and the radiographic finding of punctate calcifications in the epiphyseal cartilages (chondrodysplasia punctata, CDP). Most patients have congenital cardiac defects. The clinical course involves dramatic postnatal growth deficiency and profound global cognitive and developmental disabilities [Braverman et al., 2001 November 16 (Updated 30 January 2020)]. Most patients also develop seizures, frequent pulmonary infections, feeding and swallowing disorders (White et al., 2003; Bams-Mengerink et al., 2013; Huffnagel et al., 2013; Duker et al., 2016). Hypomyelination and cerebellar atrophy has been observed on brain magnetic resonance imaging (MRI) (Bams-Mengerink et al., 2006). Classic RCDP has a 55% mortality rate by age 12 years and death usually results from respiratory complications (Duker et al., 2019). The biochemical profile of classic RCDP1 includes markedly reduced erythrocyte Pls and high plasma PA levels (if not under dietary restriction) with essentially normal plasma very long chain fatty acids (VLCFA).

Nonclassic (mild) RCDP1 is characterized by congenital cataracts, variable skeletal defects and neurobehavioral issues (Braverman et al., 2002; Bams-Mengerink et al., 2013; Yu et al., 2013; Fallatah et al., 2020). A recent survival analysis and natural history study showed that 91% of mild RCDP1 individuals survive to adulthood and have better growth and developmental outcomes (Braverman et al., 2002; Bams-Mengerink et al., 2013; Yu et al., 2013; Duker et al., 2019; Fallatah et al., 2020). Their biochemical profile shows reduced erythrocyte Pls levels that were higher than classical RCDP, and up to 43% of healthy controls (Fallatah et al., 2020). These individuals can accumulate high PA levels on unrestricted diets.

Additionally, mild *PEX7* deficiency can result in a phenotype like Adult Refsum disease (ARD), a disorder caused by isolated accumulation of PA due to mutations in the peroxisomal enzyme, PHYH (van den Brink et al., 2003). PA is a saturated methyl-branched-chain fatty acid that is exclusively of dietary origin, obtained mainly from fat of ruminant animals that can release phytol from chlorophyll. ARD presents with retinitis pigmentosa, anosmia, peripheral neuropathy, cerebellar ataxia and hearing loss in early adulthood due to gradual accumulation of dietary PA [Wanders et al., 2006 March 20 [Updated 11 June 2015)]. Patients with ARD due to *PEX7* defects might present earlier with sensorimotor neuropathy and cataract, an overlapping feature of RCDP. These individuals usually have near normal erythrocyte Pls levels and minimal impairment in Pls synthesis but have significant elevation in plasma PA levels that could be within the range of classic ARD patients (van den Brink et al., 2003; Horn et al., 2007).

Genotype-phenotype correlations have shown that the common *PEX7* allele, p. L292X, is a founder allele in individuals of northern European descent (Braverman et al., 2000). It is a nonfunctional protein and homozygosity is associated with the classic RCDP phenotype (Braverman et al., 2002). In contrast, unique hypomorphic variants were solely linked to a milder RCDP or ARD phenotype and could be associated to residual PEX7 protein activity (Braverman et al., 2000; Braverman et al., 2002; Motley et al., 2002; Fallatah et al., 2020). There is evidence that a few of these *PEX7* hypomorphic alleles result from residual amounts of normal *PEX7* transcript and protein possibly “leaking” through splice site or 5’ UTR mutations (Braverman et al., 2002).

The overall estimated RCDP prevalence rates are between 0.5 and 0.7 cases per 100,000 births in the US and Europe, respectively (Luisman et al., 2021). RCDP can be caused by defects in 5 peroxisomal genes that result in Pls deficiency. RCDP1 accounts for 90% of cases and is caused by mutations in *PEX7,* encoding the cytosolic receptor for peroxisomal matrix proteins carrying the Peroxisomal Targeting Signal 2 (PTS2). PEX7 receptor defects impair peroxisomal import of the three known PTS2-enzymes: alkylglycerone-phosphate synthase (AGPS), phytanoyl-CoA hydroxylase (PHYH) and 3-oxoacyl-CoA thiolase or acetyl-CoA acyltransferase (ACAA1) (Braverman et al., 1997; Motley et al., 1997; Purdue et al., 1997)*.* Consequently, PEX7 deficiency results in reduced Pls synthesis (due to AGPS deficiency) and PA degradation (due to PHYH deficiency). Peroxisomal thiolase (ACAA1) deficiency does not impair the oxidation of VLCFA in humans, presumably due to availability of sterol carrier protein X (SCPx), a PTS1-enzyme with thiolase activity (Seedorf et al., 1994; Wanders et al., 1997). The RCDP phenotype results also from isolated deficiency in peroxisomal enzymes Glyceronephosphate O-acyltransferase (GNPAT) (Wanders et al., 1992; Barr et al., 1993), AGPS (Wanders et al., 1994) and fatty acyl-CoA reductase 1 (FAR1) (Buchert et al., 2014), causing RCDP2-4 respectively. RCDP5 is caused by defects in a specific domain of the peroxisomal matrix enzyme co-transporter, PEX5, that binds to PEX7 (Baroy et al., 2015).

The unique deficiency of Pls phospholipids in RCDP highlights their importance in the development and homeostasis of multiple organ systems. Acquired Pls deficiency has also been reported in common disorders including neurodegenerative, respiratory, cardiovascular and metabolic diseases that highlight the important role of Pls in a wide range of physiological functions (Braverman and Moser, 2012; Dorninger et al., 2017a; Paul, Lancaster and Meikle, 2019). Pls are ether phospholipids typically characterized by the presence of a C16:0, C18:0 or C18:1 fatty alcohol with a vinyl ether linkage at the sn-1 position, an ester bond linking mainly to polyunsaturated fatty acids (such as arachidonic acid (AA) or docosahexaenoic acid (DHA)) at the sn-2 position, and polar head groups of phospho-ethanolamine (most abundant form in the brain and many tissues) or phospho-choline at the sn-3 position of the glycerol backbone. Pls bring unique structural and functional features to membranes, and are critical components of myelin sheaths, synaptic vesicles in neuronal junctions as well as the pulmonary surfactant barrier (Brites et al., 2004; Braverman and Moser, 2012). Pls have antioxidant effects (Luoma et al., 2015; Wu et al., 2019) and are considered a valuable source of the polyunsaturated omega-3 fatty acid DHA (C22:6 n−3), an essential structural component of the human brain and retina (Hishikawa et al., 2017). Pls play a significant role in modulating cellular signaling, cholesterol biosynthesis, innate immunity and macrophage phagocytosis (Pike et al., 2002; Brodde et al., 2012; Honsho, Abe and Fujiki, 2015; Di Cara et al., 2017; Rubio et al., 2018).

Studying the neurological aspects of RCDP in terms of pathology and function is critical to understand the pathophysiological consequences of Pls deficiency in the nervous system and for determining endpoints in pre-clinical therapeutic trials. Previous *in-vivo* nervous system studies of *Pex7* null (RCDP1) and *Gnpat* null (RCDP2) mouse models showed abnormal myelination in central and peripheral nervous systems, impairment in cerebellar Purkinje cells foliation and innervation, and deficits in brain neurotransmitters and nerve conduction velocity (Brites et al., 2003; da Silva et al., 2014; Malheiro et al., 2019; Rodemer et al., 2003; Teigler et al., 2009; Dorninger et al., 2019b). *Gnpat* null mice exhibited hyperactive and stereotypic behavioral patterns, social interaction impairment as well as defects in neuromuscular transmission and neuromotor function (Dorninger et al., 2017b; Dorninger et al., 2019a; Dorninger et al., 2019b).

We previously reported the initial characterization of a hypomorphic *Pex7* mouse model that resembled a mild RCDP1 phenotype and showed early cataracts, abnormalities of lens epithelial cells and delayed skeletal ossification (Braverman et al., 2010). Full length Pex7 transcript was reduced to <5% of control due to the intronic insertion of a neo cassette. Here we used these mice to generate and characterize a *Pex7* deficient allelic mouse series representing the severe (*Pex7* ^null/null^) and milder RCDP1 phenotypes (*Pex7* ^hypo/null^ and *Pex7* ^hypo/hypo^). These *Pex7* deficient mice show graded changes in growth, survival and biochemical profiles including Pls, VLCFA, and PA levels that reflected the degree of *Pex7* deficiency. Histological assessment showed myelin reduction and Purkinje cells loss. All *Pex7* deficient mice reveal increased activity levels in the open field environment. These models help to correlate biochemical changes with disease severity, including the correlation between Pls reduction and neurotransmitter levels, and show a potential role for PA accumulation in the cerebellar phenotype. Finally, the marked reduction observed in *Pex7* transcript and protein levels in the hypomorphic mice suggest that small increases in *Pex7* transcript and protein result in large improvement in the RCDP phenotype and are useful for pre-clinical trial planning.

## 2 Materials and Methods

### 2.1 Generation, Care and Tissue Collection of *Pex7* Deficient Mouse Series

We used the previously reported *Pex7* hypomorphic mouse model (Braverman et al., 2010) B6;129S6-*Pex7* ^tm2.0Brav^ (named here as *Pex7* ^hypo/hypo^), to generate an allelic series, (B6;129S6-*Pex7* ^tm2.2Brav^) referred as *Pex7* ^null/null^ and (B6;129S6-*Pex7* ^tm2.3Brav^) referred to as *Pex7* ^hypo/null^. The *Pex7* null allele was generated by breeding CMV-driven Cre recombinase deleter mice, B6.C-Tg(CMV-cre)1Cgn/J, with *Pex7* ^hypo/hypo^ mice, in which loxP sites flanked exon 3. Four different primer sets were used to distinguish the *Pex7* ^hypo^, *Pex7* ^null^ and *Pex7* ^WT^ alleles; unique primer combinations distinguished each genotype (see Supplementary Table S1 for primer list). *Pex7* ^WT/null^ mice were bred to obtain *Pex7* ^null/null^ mice. *Pex7* ^WT/null^ females were bred with *Pex7* ^hypo/hypo^ males to generate the intermediate *Pex7* ^hypo/null^ mouse model. SNP genotyping showed a stable 87% 129S6/SvEvTac and 13% C57BL/6NCrl strain background (MaxBax, Charles River, Wilmington, MA). For PCR genotyping, ear notches were collected in 75 μl of alkaline lysis buffer (25 mM NaOH/0.2 mM Na_2_EDTA) at 95°C × 30 min for digestion followed by neutralization with 250 μl of 40 mM Tris-HCl. *Gnpat* null mice (*Gnpat* ^*tm1Just*^, MGI: 2670462) (Rodemer et al., 2003) were maintained on an outbred C57BL/6 × CD1 background. Experimental cohorts with *Gnpat* ^null/null^ and *Gnpat* ^WT/WT^ littermates were obtained by breeding *Gnpat* ^*null/WT*^ animals; genotypes were determined by PCR as described (Rodemer et al., 2003). Mice were housed in the animal facility at the RI-MUHC and the local animal facility of the Medical University of Vienna with 12 h of dark and light cycles. Animals were fed a commercial laboratory rodent diet [Teklad Global 18% Protein, Envigo, Canada (that does not have sources of PA listed)] and had free access to water. For all experiments, we used both males and females at ages 1, 4, and 12 months. Control groups were *Pex7* ^WT/WT^ and *Pex7* ^WT/hypo^ littermates for experiments involving *Pex7*-deficient mice and *Gnpat* ^WT/WT^ littermates for experiments involving *Gnpat* ^null/null^ mice. Euthanasia was performed by CO_2_ preceded by 5% isoflurane anesthesia. For gene expression, protein studies and biochemical analysis, tissues (cerebral cortex, cerebellum, heart, liver, lung, and kidney) were collected, snap frozen in liquid nitrogen and stored at −80°C until analysis. Blood samples and separation of plasma from erythrocytes was prepared as described (Fallatah et al., 2019). For histology, whole brain tissue was harvested and fixed in 10% neutral buffered formalin at 4°C × 48 h, then transferred to 70% ethanol and processed for paraffin embedding. This study was conducted under a McGill University animal care committee approved protocol (#5538) or a license by the Austrian Federal Ministry of Science and Research (BMBWF-66.009/0174-V/3b/2019).

### 2.2 Quantitative Real-Time PCR (qPCR)

Total RNA was extracted from ∼20 mg of cerebral cortex or cerebellum using TRIZOL™ (Invitrogen). For cDNA synthesis, we used 1 μg of RNA and OneScript^®^ Plus cDNA Synthesis SuperMix reverse transcriptase (Abm, G453), and qPCR with EvaGreen 2X qPCR Kit (Abm™ II) and CFX96 Touch Real-Time PCR Detection System (Bio-Rad). *Pex7* gene expression was normalized to murine hypoxanthine guanine phosphoribosyltransferase (*Hprt*) using Bio Rad software (Bio-Rad CFX Maestro). qPCR experiments were performed twice per sample; in each experiment, we used 3 biological and 2 technical replicates per genotype. Primer sets are listed in Supplementary Table S1.

### 2.3 Immunohistochemistry (IHC)/Immunofluorescence (IF)

Paraffin embedded brain sections were sagittally sectioned (7 μm) and used for protein detection. We used stainless-steel brain matrices to obtain uniform sagittal sections across different animals. Allen Mouse Brain reference Sagittal Atlas version 1, 2008 (Sunkin et al., 2013) was used as a reference to match levels of cerebral cortex sections and all sections were between levels 15–16 (0.875–1.10 mm lateral to midline). Brain sections were deparaffinized with xylene and rehydrated with decreasing concentrations of ethanol and rinsed in 1X phosphate-buffered saline (PBS). Following antigen retrieval and serum blocking, sections were incubated overnight with primary antibody. Secondary antibodies were applied, and sections processed by fluorescent labeling and mounting using ProLong Gold antifade reagent with DAPI (Invitrogen, Carlsbad, CA) for IF staining, or DAB following a Vectorstain Elite ABC kit (Vector Laboratories) and hematoxylin counterstaining for IHC staining. Images were detected using a Leica DM6000B microscope with DFC345FX camera and LASX software (Richmond Hill, Canada). Primary antibodies and concentrations used were: mouse monoclonal anti-msCalbindin-D-28K (1:200, C9848, Sigma Aldrich), rat monoclonal anti-Myelin Basic Protein antibody “MBP” (1:1000, ab7349, Abcam) and rabbit polyclonal anti-hsPEX7 (1:200, Abcam, ab167036). Secondary antibodies were: goat anti-Mouse IgG (H + L) Alexa Fluor Plus 488 (1:200, A32723, Invitrogen), Goat Anti-Rat IgG H&L HRP conjugated (1:200, ab205720, Abcam).

### 2.4 Quantification of Cerebellar Purkinje Cells

To calculate the numbers of calbindin-D28K-positive Purkinje cells, mid-sagittal cerebellar sections (0.875–1.10 mm lateral to midline) from 3-4 independent samples per genotype were analyzed. Purkinje cells were counted in three to four cerebellar slices in entirety per mouse genotype at 1, 4, and 12 months of age by two independent researchers using the cell counter plugin from ImageJ software (Schneider et al., 2012).

### 2.5 Immunoblotting

Cerebral cortex and cerebellar tissues from *Pex7* deficient mice were homogenized using TissueLyser II (QIAGEN, cat#:85300) in RIPA buffer as previously described (Argyriou et al., 2019). Primary antibodies used were: rabbit polyclonal anti-beta-Tubulin (1:17,000, Abcam, ab6046), and rabbit monoclonal anti-PEX7 (1:1000 Abcam, ab134962). Visualization of membrane was performed using an Amersham 600 gel imager. Immunoblotting and densitometric analyses were performed as previously described (Argyriou et al., 2019; Fallatah et al., 2020).

### 2.6 Behavioral Tests

#### 2.6.1 Open Field Test

The open field test was used to assess the general locomotor activity in *Pex7* deficient mice as described (Fallatah et al., 2019). For activity measures, we evaluated the total distance traveled in meters and activity time in 300 s. These parameters were analyzed using Any-Maze software (Stoelting Co., IL, United States).

#### 2.6.2 Noldus CatWalk Gait Analysis

To evaluate gait patterns of *Pex7* deficient mice, we utilized the CatWalk gait analysis system (Noldus information technology). The mice were allowed to freely cross the pressure-sensitive plate of the CatWalk. Paw prints were labeled [right-fore (RF), right-hind (RH), left-fore (LF), left-hind (LH)] and analyzed using CatWalk software. The mice were trained the first day followed by 3 days of experimental sessions. Gait parameters were recorded and averaged over the 4 successful trials per session per day. A successful complete trial was defined as uninterrupted tracks with at least 4 cycles of complete steps and with speed variation less than 25% between the mice. Any mouse that did not complete 4 cycles in 15 min was excluded from the analysis.

#### 2.6.3 Forced Alternation Test (Y-Maze)

Forced alternation tests were performed using a symmetrical Y-maze containing three identical arms and made of a grey acrylic bottom and walls (Stoelting Co., Wood Dale, IL). Each Y-maze arm was 50 cm long, 10 cm wide, and 20 cm high. The mice were trained for 3 days followed by 3 days of the experimental test. The test used a 5-min sample trial (first test) followed by a 5-min retrieval trial (second test). In the first test, the mouse was placed at the end of the start arm, facing the wall and away from the center. The mouse was then allowed to freely explore only two arms of the Y-maze, while entry into the third arm was blocked. After the sample trial, the mouse was returned to its home cage for 30 min. In the second test, the block in arm 3 was removed; the mouse was again placed in the start arm and allowed to explore all three arms of the maze. An arm entry was recorded when 90% of a mouse’s body entered the arm. Both tests were recorded by an overhead USB camera (model 60531, Stoelting CO., Wood Dale, IL, United States). Footage was analyzed by an automated tracking system (Any-maze Video Tracking Software, Stoelting CO. Wood Dale, IL, United States) for the number of entries and the percentage of time spent in each arm. A successful response considered to be correct when the number of entries and the time spent in the novel arm are significantly higher than other arms. Each mouse was subjected to 5 consecutive trials in a session per day. Any mouse with less than three arm entries in the first minute of the second test was excluded from the analysis.

### 2.7 LC-MS/MS Analysis for PlsEtn and C26:0-LPC Levels

Reagents used were authentic Pls standards, tetradeuterated internal standards 26:0-D4 lysoPC (Avanti Polar Lipids, Alabaster, Alabama), 16:0-D4 lyso PAF (Cayman Chemical Company, Ann Arbor, Michigan) and HPLC grade solvents (methanol, acetonitrile, chloroform, water) (Fisher Scientific, Waltham, MA), formic acid (Honeywell Fluka), ammonium formate (Sigma-Aldrich), and PBS (Thermo Fisher Scientific, Waltham, MA). LC-MS/MS analysis was performed in blood and tissues from *Pex7* deficient mice as previously described (Fallatah et al., 2019). PlsEtn were detected by multiple reaction monitoring (MRM) transitions representing fragmentation of [M + H] + species to m/z 339, 361, 385, 389, and 313 for compounds with 18:1, 20:4.22:6, 22:4, and 16:0, at the sn-2 position, respectively. Lysophosphatidyl choline (LPC) species were detected by MRM transitions representing fragmentation of [M + H] + species to m/z 104.

### 2.8 GC/MS Analysis for PA and DHA

Analysis was done in plasma, cerebral cortex and cerebellum from *Pex7* and *Gnpat* deficient mice as well as rodent chow using standard methods in the Peroxisome Disease Laboratory, Kennedy Krieger Institute, Baltimore Maryland (Dacremont et al., 1995; Dacremont and Vincent, 1995; Krauß et al., 2017).

### 2.9 High-Performance Liquid Chromatography Analysis for Brain Neurotransmitters

Analysis was performed on mice half cerebrum tissues without cerebellum using High-Performance Liquid Chromatography (HPLC) with electrochemical detection (monoamines) or fluorometric detection (amino acids) as previously reported (Hörtnagl et al., 1991; Peneder et al., 2011; Dorninger et al., 2019b).

### 2.10 Statistical Analysis

Data analysis was done using the GraphPad Prism software package (version 9.0) (GraphPad Software, La Jolla, United States). Statistical analysis was performed using one way or two-way analysis of variance (ANOVA) followed by appropriate correction tests for multiple comparisons. Simple linear regression was performed for correlation analysis between specific variables. Data are shown as the mean ± SD and statistical significance was set at *p* < 0.05. Since we did not observe any significant differences between male and female animals in biochemical and behavioral results, the data obtained from both genders were combined in the final statistical analysis.

## 3 Results

In this study, an allelic series of *Pex7* deficient mice were generated to investigate the broad phenotypic spectrum of RCDP1. These mice include 2 hypomorphic models: a previously reported homozygous, *Pex7* ^hypo/hypo^ (Braverman et al., 2010) and a novel compound heterozygous, *Pex7* ^hypo/null^, resembling milder RCDP1. The third model, *Pex7* ^null/null^_,_ represents severe RCDP1.

### 3.1 *Pex7* Transcript and Protein Levels are Extremely Reduced in Brain Tissues From *Pex7* Deficient Mice

The *Pex7* hypomorphic mouse model contains a *neo* cassette in reverse orientation in intron 2 and lox sites flanking exon 3 (Braverman et al., 2010). The intronic neo cassette reduces full length transcript levels and the lox sites were used to generate the null allele; thus all mice were generated from the same construct (Meyers et al., 1998). To determine *Pex7* transcript levels in our *Pex7* deficient mouse series, we performed quantitative RT-PCR analysis in cerebral cortex, cerebellum, liver, lung and kidney tissues. The average *Pex7* gene expression levels for *Pex7* ^hypo/hypo^ and *Pex7* ^hypo/null^, were 0.394 ± 0.3% and 0.174 ± 0.1% of wild type *Pex7* transcript, respectively. Pex7 transcript was not detected in any tissues from *Pex7* ^null/null^ (Table 1).

**TABLE 1:** *Pex7* transcript levels according to the genotype severity of *Pex7* deficient mice.

*Pex7* genotype	*Pex7*				*Hprt*			
	WT/WT	hypo/hypo	hypo/null	null/null	WT/WT	hypo/hypo	hypo/null	null/null
**Cerebral cortex**								
Relative expression^a^ (SEM)^b^	100.0 (32.61)	0.061 (0.015)	0.016 (0.011)	–	–	–	–	–
Mean Cq^c^ (SEM)	23.21 (0.286)	33.37 (0.311)	35.35 (0.885)	–	18.77 (0.373)	18.26 (0.15416)	18.28 (0.495)	18.71 (0.411)
**Cerebellum**								
Relative expression (SEM)	100.0 (17.49)	0.181 (0.030)	0.081 (0.039)	–	–	–	–	–
Mean Cq (SEM)	23.85 (0.186)	32.64 (0.166)	35.10 (0.669)	–	17.95 (0.232)	17.59 (0.348)	19.16 (0.235)	18.51 (0.189)
**Liver**								
Relative expression (SEM)	100.0 (43.98)	0.529 (0.179)	0.245 (0.096)	–	–	–	–	–
Mean Cq (SEM)	24.09 (0.459)	32.04 (0.321)	33.98 (0.422)	–	20.22 (0.438)	20.60 (0.368)	21.43 (0.372)	21.06 (0.244)
**Lung**								
Relative expression (SEM)	100.0 (16.97)	0.566 (0.193)	0.273 (0.076)	–	–	–	–	–
Mean Cq (SEM)	26.14 (0.191)	33.91 (0.305)	36.95 (0.342)	–	20.68 (0.155)	20.90 (0.137)	22.51 (0.204)	21.19 (0.531)
**Kidney**								
Relative expression (SEM)	100.0 (23.89)	0.633 (0.134)	0.257 (0.056)	–	–	–	–	–
Mean Cq (SEM)	25.26 (0.227)	33.11 (0.204)	33.77 (0.196)	–	22.96 (0.259)	23.49 (0.225)	22.86 (0.248)	22.81 (0.275)

a Relative expression of Pex7 to Hprt is reported as the percentage of Pex7 wild type expression. Each tissue and genotype represent the means of 3 mice performed in duplicates.

b SEM (the standard error of mean) which is used as a measure of precision for the estimated mean.

c Cq (the quantification cycle value of an amplification reaction) is defined as the fractional number of cycles that were needed for the fluorescence to reach a quantification threshold.

**FIGURE 1:**
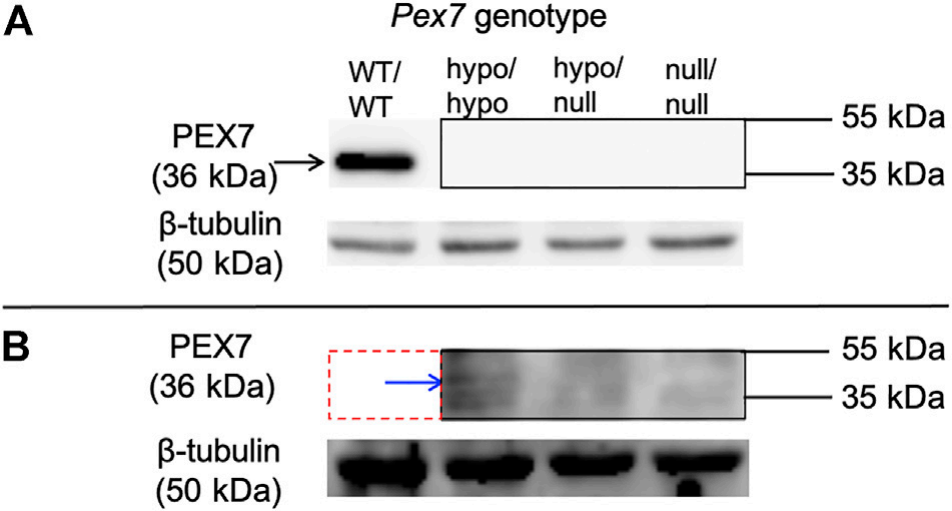
PEX7 protein levels in the brain are undetectable in *Pex7* ^null/null^ or *Pex7* ^hypo/null^ and barely detectable in *Pex7* ^hypo/hypo^ mice. Immunoblotting of the cerebral cortex tissue lysates showing that PEX7 protein is not detected in any of the *Pex7* deficient models at the amount of 40 μg of protein and 10 min exposure time **(A)**. However, at protein quantity of 100 μg and exposure time of 20 min, small amounts of PEX7 protein are only detected in *Pex7* ^hypo/hypo^ mice (blue arrow) **(B)**, the red dotted line represents the omitted *Pex7* ^WT/WT^ lane due to overexposure. β-tubulin was used as a loading control.

To investigate the endogenous amounts of PEX7 protein in *Pex7* deficient mice, immunoblot analysis using PEX7 antiserum was performed on brain tissue homogenates. PEX7 protein could not be detected in the brain tissue homogenates from *Pex7* ^hypo/null^ or *Pex7* ^null/null^ mice. However, with higher protein quantity and longer exposure, traces of PEX7 protein were detected in *Pex7* ^hypo/hypo^ mice (Figure 1).

### 3.2 PEX7 Expression Is Reduced in the Cerebellar Tissue From *Pex7* Deficient Mice

**FIGURE 2:**
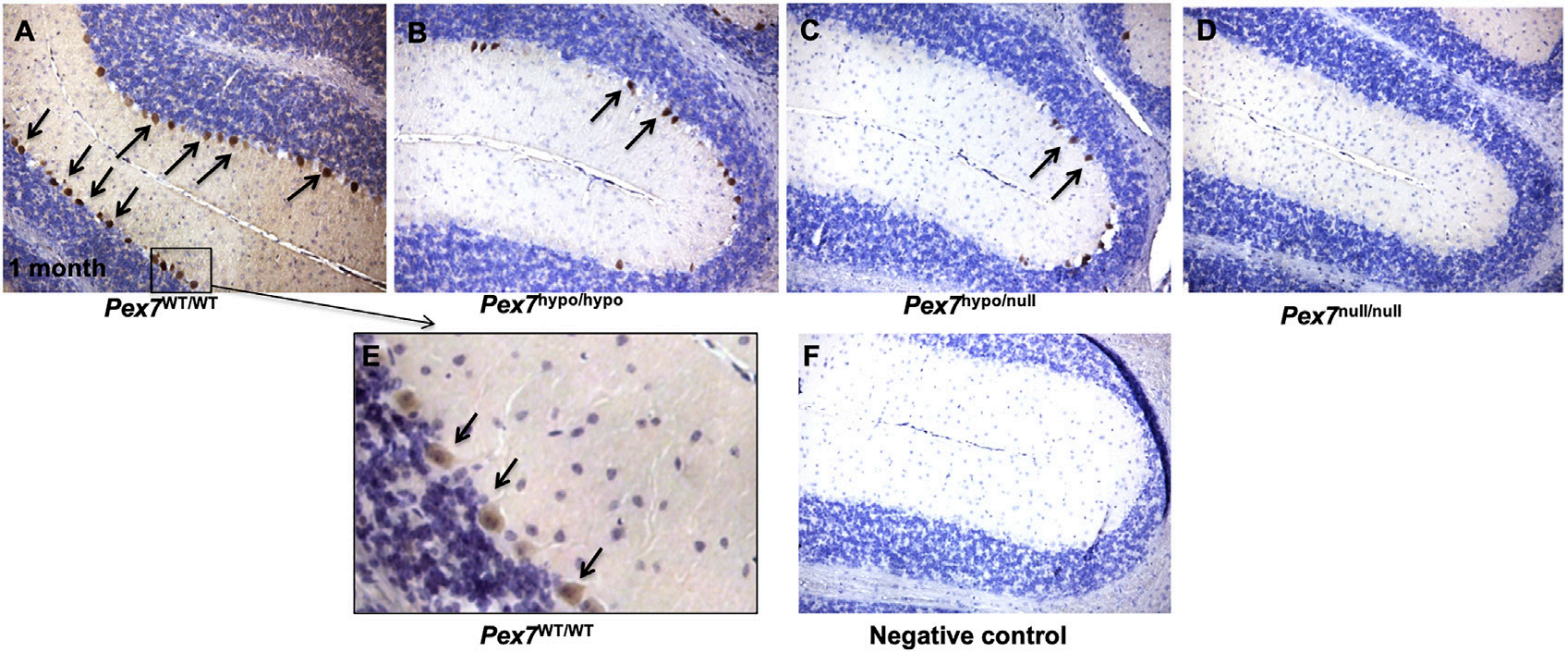
PEX7 protein expression in cerebellar tissues from 1 month old *Pex7* deficient mice. A highly concentrated PEX7 expression was observed in the cerebellar Purkinje cells of *Pex7* ^*WT/WT*^ (**(A, E)** higher magnification). However, the cerebellar Purkinje cell sections from *Pex7* ^hypo/hypo^ Pex7^hypo/null^ **(B, C)** display a reduction in PEX7 expression. PEX7 expression was entirely absent in the cerebellar Purkinje cells of *Pex7* ^null/null^ **(D)**. Negative control includes *Pex7* ^WT/WT^ section without primary PEX7 antibody **(F)**. Slides were counterstained with hematoxylin (Scale bar = 100 μm), (*n* = 3 per genotype). Sections from the same mice at 1 month of age were stained with anti-Calbindin to determine cerebellar Purkinje cells number (see Figure 8).

The distribution of PEX7 was determined by IHC. PEX7 expression was markedly localized to cerebellar Purkinje cells. Evaluation of cerebellar tissue sections of *Pex7* ^hypo/hypo^ and *Pex7* ^hypo/null^ at 1 month of age revealed a global reduction in PEX7 expression within Purkinje cells. PEX7 expression was completely undetectable in cerebellar Purkinje cells of the *Pex7* ^null/null^ mice (Figure 2). These observations suggested that PEX7 is highly expressed in Purkinje cells and thus could be critical to their normal physiological function.

### 3.3 *Pex7* Genotype Correlates With Survival Rate and Weight Gain

**FIGURE 3:**
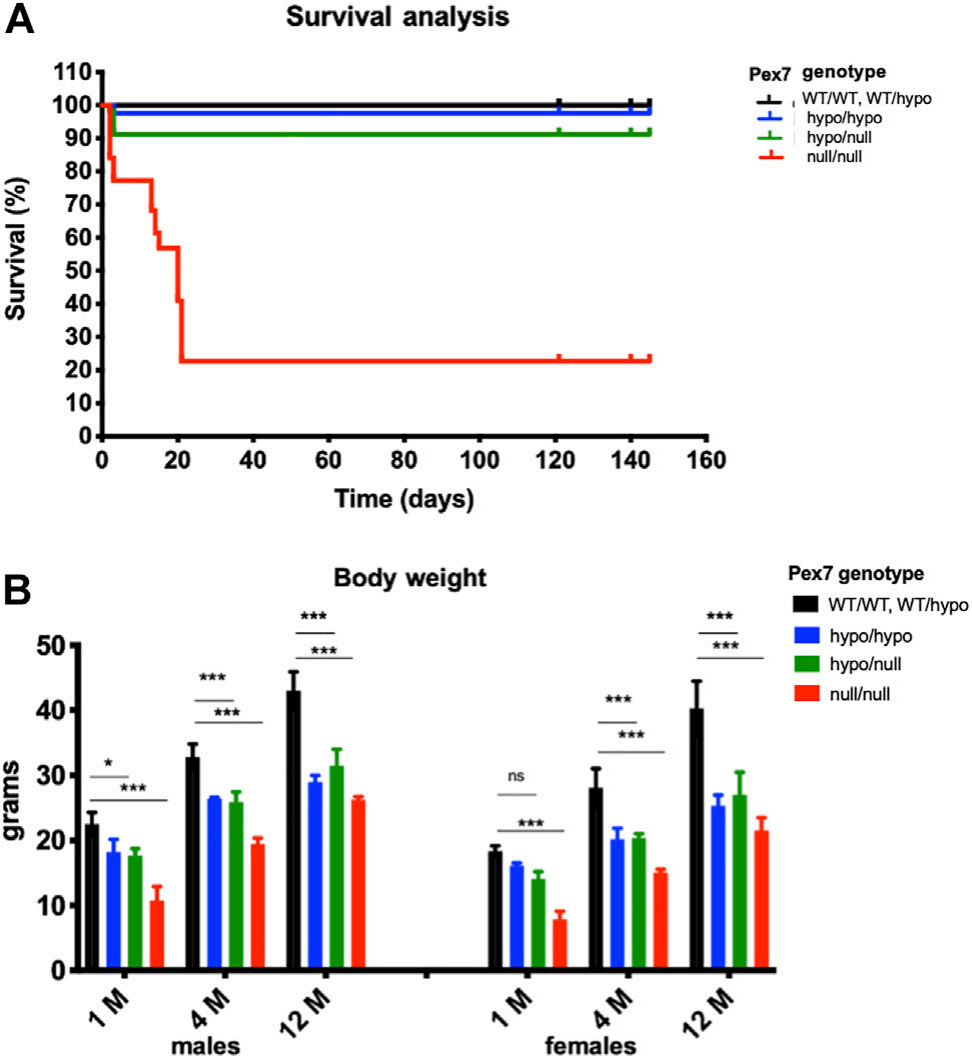
*Pex7* genotype correlates with survival rates and weight gain. Kaplan–Meier plots showing the percentage of survival of *Pex7* deficient mice (*n* = 41–60 per genotype). Analyzing the survival data by log rank tests showed significant differences between genotypes (*p* < 0.001) in accordance to the severity of *Pex7* genotype **(A)**. Graded and significant reduction in body weight according to *Pex7* genotype severity is found in *Pex7* mutants compared to controls at 1, 4, and 12 months (M) of age (*n* = 10–12 per genotype) **(B)**. ns: nonsignificant, *p < 0.05, **p < 0.01, ***p < 0.001, ****p < 0.0001.

Survival data of *Pex7* deficient mice showed survival rates of 100% and 91.2% in *Pex7* ^hypo/hypo^ and *Pex7* ^hypo/null^, respectively, over the 150 days observation period. However, only 22.7% of the *Pex7* ^null/null^ mice survived beyond 21 days. Around 45% of *Pex7* ^null/null^ pups died within the first 3 days of life and 32% died before weaning (Figure 3A). Close observation of the latter showed poor weight gain in general and reduced activity a few days before death; the exact cause of death remains unknown. The *Pex7* ^hypo/hypo^ and *Pex7* ^hypo/null^ animals weighed 70%–80% and 67%–78% of their control littermates respectively. However, the *Pex7* ^null/null^ mice weighed only 47%–53% of littermate controls. Measurements were performed at 1, 4 and 12 months of age (Figure 3B). Although body length was not measured, the mice appeared smaller in size, and their length appeared proportional to the reduction in weight.

### 3.4 *Pex7* Genotype Correlates With Peroxisome Metabolite Levels

#### 3.4.1 Phosphatidylethanolamine Plasmalogens (PlsEtn) Levels

**FIGURE 4:**
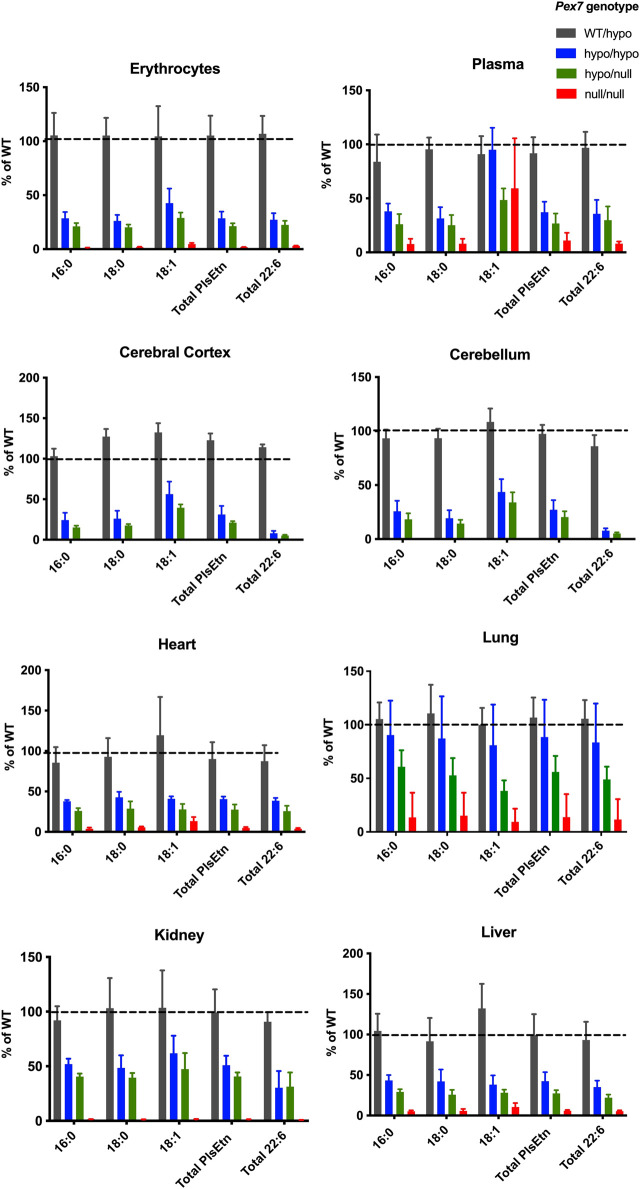
*Pex7* genotype correlates with PlsEtn levels. Pls levels [reported as the percentage of wild type (% of WT)] are significantly decreased in all tissues studied in accordance to the severity of the genotype of *Pex7* deficient mice (*p < 0.0001*, *n* = 4–6 per genotype). The C16:0, C18:0, and C18:1 represent the sum of individual measurements of the PlsEtn species with C16:0, C18:0, or C18:1 fatty alcohol at the *sn-*1 position. Total PlsEtn refers to the sum of these species and the total C22:6 represents the sum of C16:0, C18:0, and C18:1 Pls containing C22:6 fatty acid side chains at the *sn-*2 position.

We measured PlsEtn that contain C16:0, C18:0, and C18:1 fatty alcohols at sn*-*1 in plasma, erythrocytes and tissues (cerebral cortex, cerebellum, lung, heart, liver and kidney) from the *Pex7* deficient mouse series using LC-MS/MS. In *Pex7* ^hypo/hypo^ and *Pex7* ^hypo/null^, the total PlsEtn levels were reduced to 30%–69% and 20%–50% of wild type controls, respectively. PlsEtn were undetectable in cortical or cerebellar brain regions of *Pex7* ^null/null^ mice, and markedly decreased in blood and peripheral tissues to 5%–15% of wild type littermates (Figure 4). Pls species containing DHA (C22:6) at *sn-2* showed graded and profound deficiency in all tissues evaluated according to genotype severity.

In control mice (*Pex7* ^WT/WT^ and *Pex7* ^WT/hypo^), we observed that distribution and quantity of PlsEtn subspecies were tissue dependent. The highest amount of Pls was found in the brain followed by heart, lung, erythrocyte and kidney while the lowest Pls quantity was observed in liver and plasma (Supplementary Figure S1). Among PlsEtn species, C18:0 was the most abundant subclass in all tissues examined except lung which was enriched in C16:0. In contrast, C18:1 was the least abundant Pls subspecies in all peripheral tissues evaluated except lung in *Pex7* deficient mice. There were no significant differences in terms of distribution or quantity of total Pls between the two control groups, *Pex7* ^*WT/WT*^ and *Pex7* ^*WT/hypo*^ (Supplementary Figure S1).

#### 3.4.2 C26:0-LPC Levels

**FIGURE 5:**
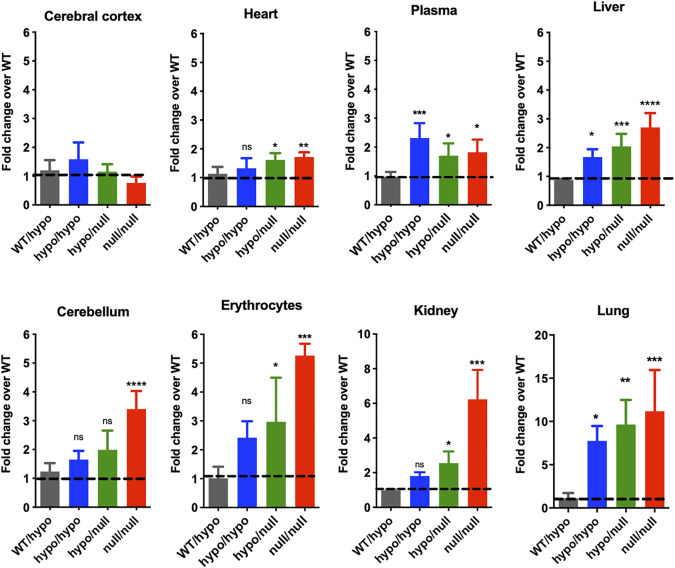
*Pex7* genotype correlates with C26:0-LPC (VLCFA) levels. Elevations in C26:0-LPC represented as fold change over wild-type (WT) are detected in the blood and several tissues of 4-month-old *Pex7* deficient mice. No significant increase in C26:0-LPC levels was found in the cerebral cortex of *Pex7* deficient mice (*n* = 4–6 per genotype). The dotted line represents the C26:0-LPC levels in *Pex7* ^WT/WT^ mice. ns: nonsignificant, *p < 0.05, **p < 0.01, ***p < 0.001, ****p < 0.0001.

We examined the levels of C26:0-LPC, a sensitive measure of the VLCFA C26:0 content, in blood and tissues from *Pex7* deficient models using LC-MS/MS. Interestingly, these results showed tissue dependent elevations in C26:0-LPC and a direct correlation between *Pex7* genotype and C26:0-LPC levels. C26:0-LPC levels were highest in lung, but also increased in blood and peripheral tissues (kidney, liver, heart). In contrast, C26:0-LPC levels were normal in cerebral cortex. Specifically, *Pex7* ^hypo/hypo^ showed a 2-7-fold increase in C26:0-LPC levels over *Pex7* controls in lung, liver and blood, whereas *Pex7* ^hypo/null^ exhibited a 2–9-fold increase in all peripheral tissues. However, *Pex7* ^null/null^ mice displayed a more marked elevation of 2–11-fold change over *Pex7* wild type controls in all peripheral tissues as well as the cerebellar region of the brain (Figure 5).

#### 3.4.3 Phytanic Acid Levels

**FIGURE 6:**
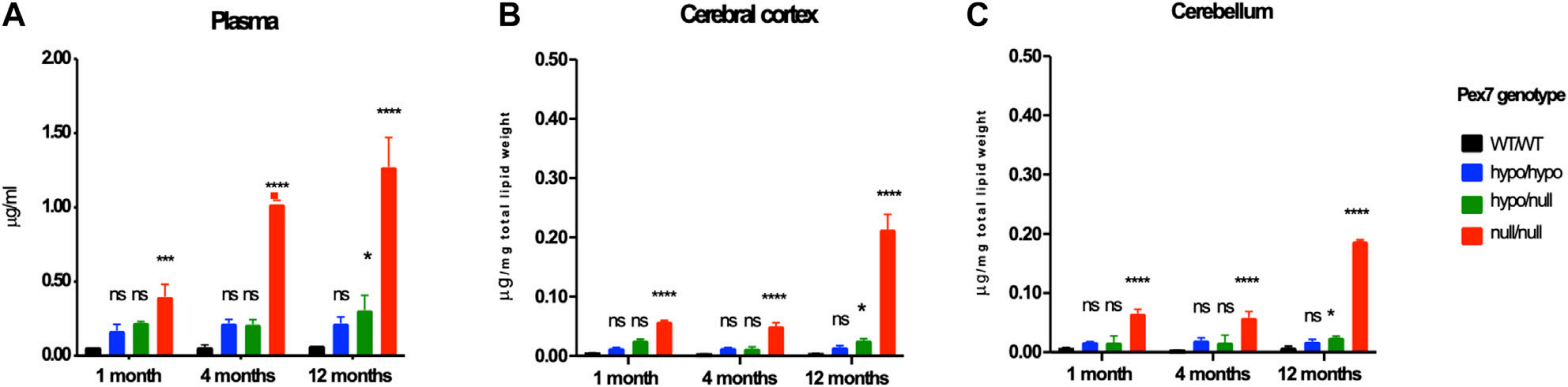
*Pex7* genotype correlates with PA levels. Graded accumulation of PA levels is detected in the plasma **(A)**, cerebral cortex **(B)** and cerebellum **(C)** of *Pex7* deficient mouse models compared to WT controls. (*n* = 2–4 per genotype). ns: nonsignificant, *p < 0.05, **p < 0.01, ***p < 0.001, ****p < 0.0001.

Phytanic Acid (PA) levels were examined by GC/MS in plasma, cerebral cortex and cerebellar tissues from the *Pex7* deficient mice at 1, 4, and 12 months of age. While PA was not elevated in plasma or brain tissues from *Pex7* ^hypo/hypo^ mice, a minor and significant elevation of PA levels was found in *Pex7* ^hypo/null^ mice only at 12 months of age. A much higher accumulation of PA was detected in the *Pex7* ^null/null^ mice with the highest elevation observed in plasma compared to brain tissues (Figure 6). There is a small amount of PA (0.373 mg/100 g) detected in standard mouse chow. The total free and esterified phytol in standard mouse chow is 1.87 mg/100 g weight of mouse chow. Additionally, there is very little phytol in the chlorophyll fraction of mouse chow. We concluded that even these small amounts of PA in rodent diet could lead to PA toxicity over time in *Pex7* deficient mice.

### 3.5 Peroxisome Metabolites in *Gnpat* ^null/null^ Mice

*Pex7* deficiency results in a complex biochemical phenotype of Pls and DHA deficiencies in addition to elevations in VLCFA and PA (Brites et al., 2003; Brites et al., 2009; Braverman et al., 2010). To determine whether these biochemical phenotypes were specific for RCDP1 due *Pex7* deficiency or associated with other RCDP types, we concurrently examined the biochemical profiles of plasma and brain tissues from 4-month-old *Gnpat* ^null/null^ mice. We found that *Gnpat* ^null/null^ mice were deficient in total PlsEtn and DHA levels. No accumulation of either C26:0-LPC or PA levels was found in plasma or brain tissues from *Gnpat* ^null/null^ mice compared to their littermate controls (Supplementary Figure S2).

### 3.6 Histopathology of Cortical and Cerebellar Brain Regions of *Pex7* Deficient Mice

Hematoxylin and eosin staining of cortical brain sagittal sections did not show any gross anatomical differences between *Pex7* deficient mice and their littermate controls at 4 months of age (data not shown). However, a reduction in the number of Purkinje cells on cerebellar sagittal sections was observed in *Pex7* ^null/null^ mice and investigated further.

#### 3.6.1 Cerebellar Purkinje Cells Number and Morphology

**FIGURE 7:**
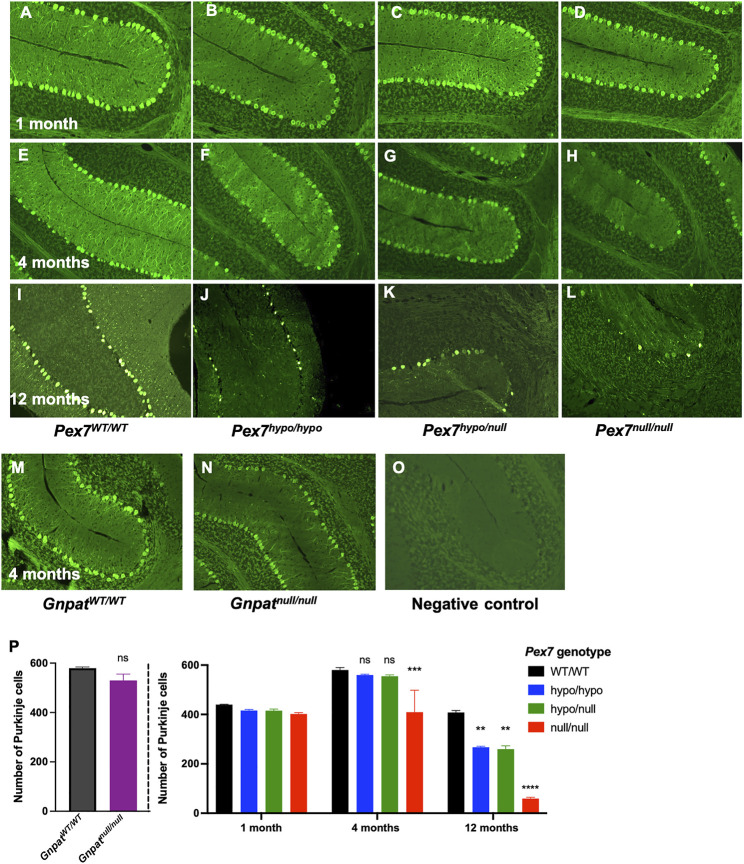
Progressive cerebellar Purkinje cells loss over time in *Pex7* deficient mice. Sagittal sections of cerebellum showed loss of Purkinje cells in the cerebellum of *Pex7* deficient mice at 4 **(E–H)** and 12 months of age **(I–L)** using anti-Calbindin Immunofluorescence staining Negative control includes *Pex7* ^WT/WT^ section without primary Calbindin antibody **(O)**. No loss of Purkinje cells in *Pex7* deficient mice at 1 month of age **(A–D)** or in *Gnpat* ^null/null^ mice **(M, N)**. Quantification of Purkinje cells in *Pex7* and *Gnpat* deficient mice in comparison to their littermate controls revealed significant decrease in Purkinje cells number in *Pex7* deficient mice **(P)**. The severe model (*Pex7* ^null/null^ mice) showed the most significant decrease in Purkinje cells numbers, with the lowest number of Purkinje cells at 12 months of age using anti-Calbindin Immunofluorescence staining. *p < 0.05, **p < 0.01, ***p < 0.001 (Scale bar = 100 μm), (*n* = 3-4 per genotype).

To further examine and quantify Purkinje cells, we performed IHC using an antibody directed to Purkinje cells (Calbindin-D28K) in the cerebellar tissues from *Pex7* deficient mice at 1, 4, and 12 months of age (Figure 7). We observed normal Purkinje cells morphology with reduced intensity of Calbindin staining in *Pex7* deficient mice. At 1 month of age, there were no differences in Purkinje cells quantity between *Pex7* deficient mice and their littermate controls (Figure 7A–D). As early as 4 months of age, *Pex7* ^null/null^ mice showed a significant loss of Purkinje cells (Figure 7E–H). In contrast, *Pex7* ^hypo/hypo^ and *Pex7* ^hypo/null^ animals had a significant reduction in Purkinje cells number only at 12 months of age (Figure 7I–L). In order to determine whether Purkinje cells loss is due to Pls deficiency, accumulated PA [since Purkinje cells loss was documented in the ARD mice fed with 0.1% and 0.25% phytol diet (Ferdinandusse et al., 2008)] or a combination of both, we evaluated cerebellar samples from the *Gnpat* ^null/null^ mice at 4 months of age. There was no significant difference in the number of Purkinje cells in *Gnpat* ^null/null^ mice compared to their wild-type controls (Figure 7M,N). These results suggested that Purkinje cells loss in *Pex7* deficient mice is due to either PA accumulation alone, or the combination of Pls deficiency and elevated PA in this model.

#### 3.6.2 Myelin Content

**FIGURE 8:**
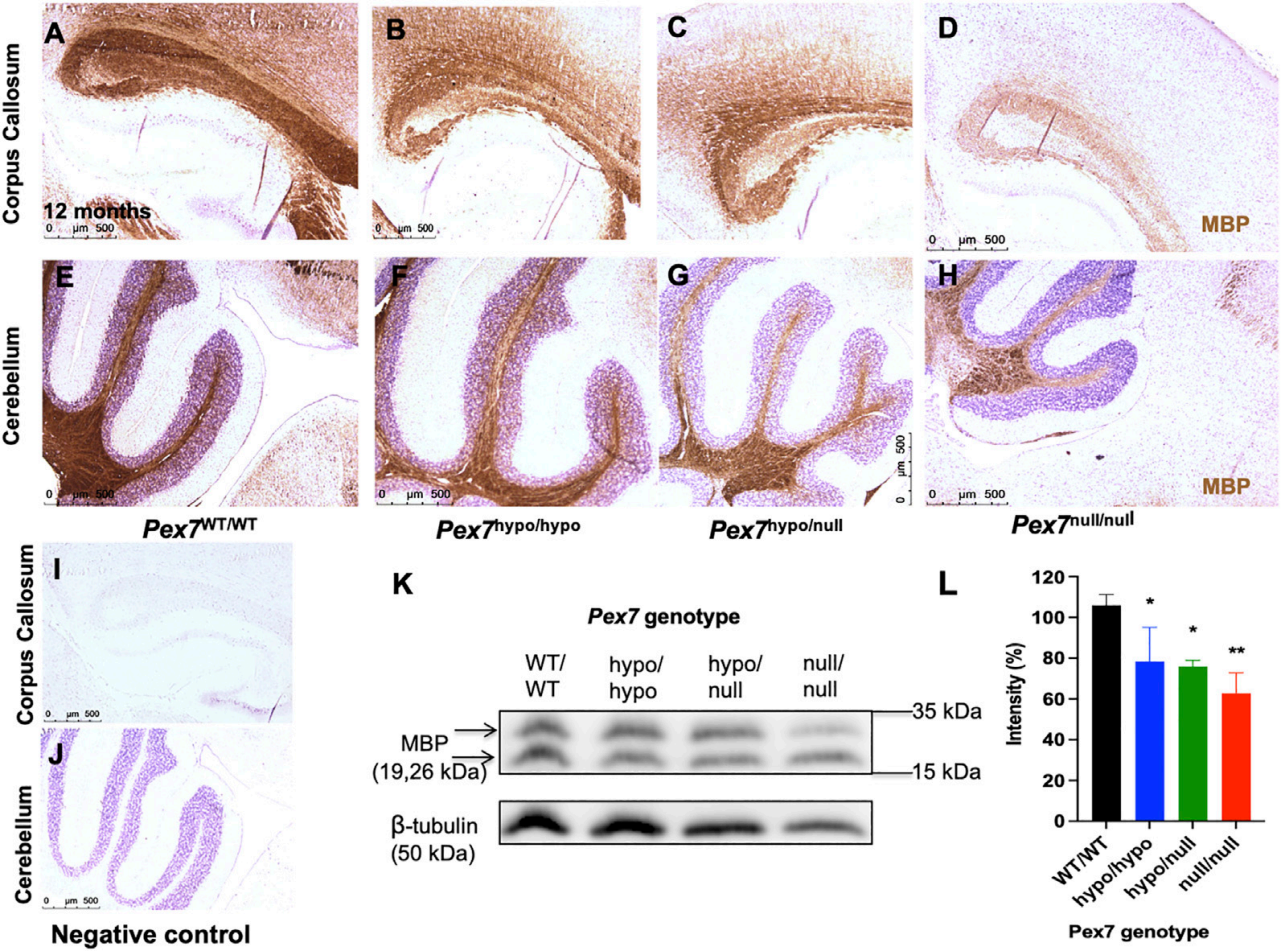
*Pex7* genotype correlates with the myelin content in brain tissues from 12-month-old *Pex7* deficient mice. Sagittal sections of brain tissues: corpus callosum **(A–D)** and cerebellum **(E–H)** show graded reduction in the levels of anti-MBP immunohistochemical staining in 12-month-old *Pex7* deficient mice compared to their littermate controls. Slides were counterstained with hematoxylin **(I, J)** (Scale bar = 500 μm). Graded reduction in the amount of MBP protein levels was found in the immunoblot and densitometric analysis of cerebellar homogenates from *Pex7* deficient mice at 12 months of age **(K, L)**. (*n* = 3 per genotype).

Since Pls is a critical component of myelin and myelin deficits were previously reported in *Gnpat* ^null/null^ mice (Rodemer et al., 2003; Teigler et al., 2009; Malheiro et al., 2019), we evaluated myelin content by IHC using MBP antibody. At 4 months of age, *Pex7* ^hypo/hypo^ and *Pex7* ^hypo/null^ mice had normal myelination in the corpus callosum and cerebellum, however, reduced MBP staining was observed in cerebellar white matter and corpus callosum in the *Pex7* ^null/null^ (data not shown). At 12 months of age, there was a slight reduction in MBP in the corpus callosum and cerebellar white matter of *Pex7* ^hypo/hypo^ and *Pex7* ^hypo/null^ mice, and an even more pronounced reduction in *Pex7* ^null/null^ mice (Figures 8A–J). The MBP protein levels of 12-month-old *Pex7* deficient mice were also decreased by immunoblot analysis in a graded fashion according to the genotype (Figures 8K,L).

### 3.7 Behavioral Assessments in *Pex7* Deficient Mice

#### 3.7.1 Increased Baseline Locomotor Activity of *Pex7* Deficient Mice in the Open Field Environment

**FIGURE 9:**
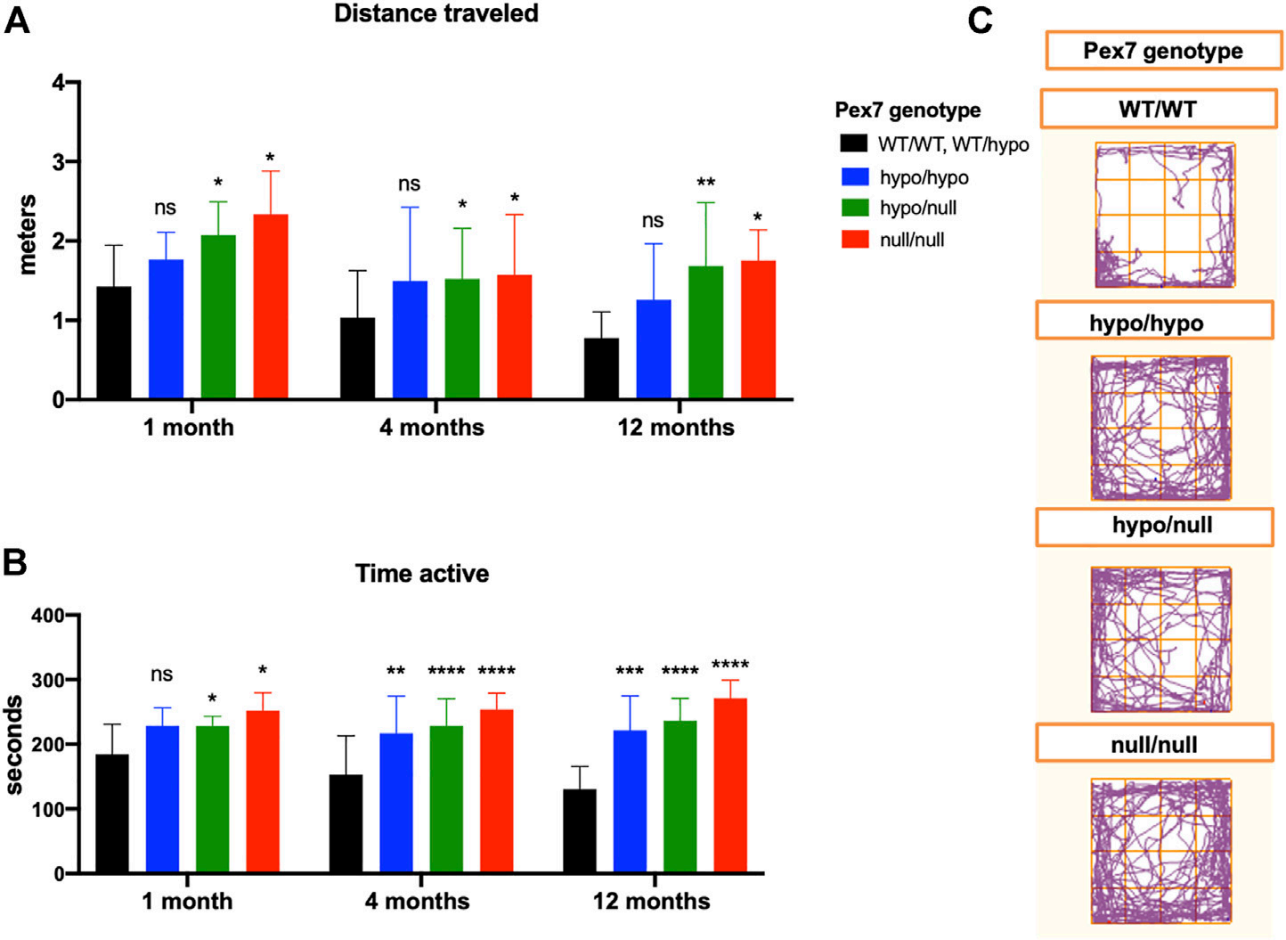
The hyperactivity behavior of *Pex7* deficient mice in the open field environment. *Pex7* deficient mice showed significant increase in activity levels measured by distance traveled **(A)** and activity time **(B)** at different ages in comparison to controls (*Pex7* ^WT/WT^ and *Pex7* ^WT/hypo^). Representative images of track blots recorded during the open field test of *Pex7* deficient mice and their littermate controls **(C)**. Bars represent group means ± SD. ns: nonsignificant, *p < 0.05, **p < 0.01, ***p < 0.001, ****p < 0.0001 (*n* = 12–36 per genotype).

We performed the open field test to assess the baseline locomotive function of *Pex7* deficient mice in response to a novel environment. In comparison to littermate controls, all *Pex7* deficient mice exhibited increased activity levels in the open field measured by increased total distance traveled and movement (activity) time. This hyperactive behavior was persistent at 1, 4, and 12 months of age in the *Pex7* deficient mouse series and the overall data trended towards a negative correlation between severity of *Pex7* genotype and activity. In fact, the *Pex7* ^null/null^ showed the highest activity levels compared to *Pex7* hypomorphic models (Figure 9).

#### 3.7.2 Late Onset Cerebellar Ataxia was Only Evident in the *Pex7* ^null/null^

PA accumulation and Purkinje cells loss within the cerebellum are often associated with the clinical finding of gait ataxia (Ferdinandusse et al., 2008). We have identified both cerebellar Purkinje cells drop out and increases in plasma and brain PA levels in *Pex7* deficient mice. However, only *Pex7* ^null/null^ mice displayed unsteady gait that was clinically evident at the age of 12 months. Considering these findings, we further analyzed the gait pattern and parameters in *Pex7* deficient mice using the CatWalk XT gait analysis system. The 12-month-old *Pex7* ^null/null^ mice showed abnormal gait parameters in the form of reduced base of support of the hind paws. No significant differences were found in any of the gait parameters analyzed in the *Pex7* ^hypo/null^ model compared to their littermate controls (Supplementary Figure S3A).

#### 3.7.3 Working Memory and Exploratory Behavior Were Unaffected in *Pex7* Deficient Mice

To evaluate working memory and exploration of new environment in *Pex7* deficient mice, we performed the forced alternation of a 3-arm Y maze test that consists of 2 test trials with a 30-min interval between the 2 trials. We measured the number of entries of each arm and percent time spent in the novel arm. No significant difference was observed between *Pex7* deficient mice and their littermate controls in the percent of time spent in the novel arm. However, *Pex7* deficient mice showed increased number of entries into each arm compared to littermate controls, which reflects the higher activity levels in these mice that also shown in the open field test (Supplementary Figures S3B-D).

### 3.8 Reduced Brain Neurotransmitter Levels in *Pex7* Deficient Mice

**FIGURE 10:**
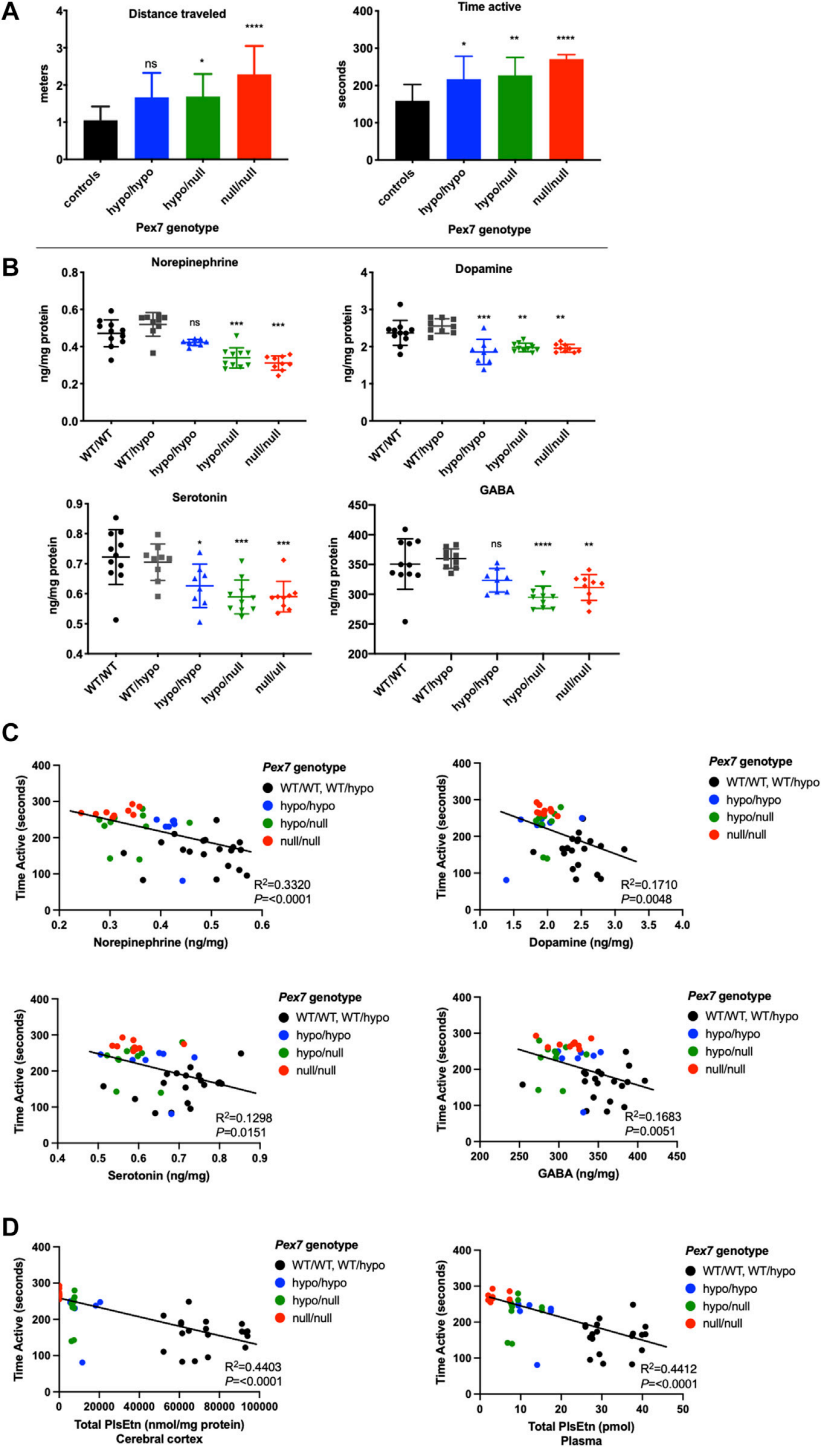
Brain neurotransmitter deficits correlate with activity levels in *Pex7* deficient mice. A significant increase in activity levels of a subset of *Pex7* deficient mice is confirmed before neurotransmitters analysis **(A)**. A significant reduction in dopamine, serotonin, norepinephrine and GABA neurotransmitters is observed in cortical brain tissues from *Pex7* deficient mice compared to littermate controls (*Pex7* ^WT/WT^ and *Pex7* ^WT/hypo^) **(B)**. The hyperactive phenotype in *Pex7* deficient mice is inversely correlated with brain neurotransmitter levels **(C)** and total PlsEtn levels in cerebral cortex as well as plasma **(D)**. Bars represent group means ± SD. ns: nonsignificant, *p < 0.05, **p < 0.01, ***p < 0.001, ****p < 0.0001 (*n* = 8–10 per genotype).

HPLC analysis was performed to examine the levels of the major monoamine neurotransmitters dopamine (DA), norepinephrine (NE) and serotonin (5-hydroxytryptamine; 5-HT) in addition to the amino acid neurotransmitter gamma aminobutyric acid (GABA) in whole brain tissue homogenates from *Pex7* deficient mice. Overall, we found a trend toward reduction in the mean levels of brain neurotransmitters across all genotypes of the *Pex7* deficient mouse series. The deficiency in DA and 5-HT levels was robust and significant in all *Pex7* deficient mice and the reduction of NE and GABA was significant in *Pex7* ^hypo/null^ and *Pex7* ^null/null^ mice. We also observed a tendency toward gradual decrease in the levels of NE and 5-HT from mild to severe *Pex7* deficient mice (Figure 10B).

Additionally, we measured the baseline levels of metabolites derived from monoamine neurotransmitters in whole brain tissue homogenates from *Pex7* deficient mice and calculated the ratio of the metabolite to its respective transmitter. This ratio reflects the turnover of monoamine neurotransmitters. No significant differences were observed between *Pex7* deficient mice and their littermate controls for the following metabolites: 5-hydroxyindoleacetic acid (5-HIAA), the main metabolite of 5-HT, 3,4-dihydroxyphenylacetic acid (DOPAC) and homovanillic acid (HVA), the primary metabolites for DA. Investigation of the ratio of the metabolites for the monoamine neurotransmitters revealed a significantly higher DOPAC/DA and 5-HIAA/5-HT ratios in *Pex7* ^hypo/hypo^ and *Pex7* ^null/null^ while the HVA/DA ratio was significantly higher only in *Pex7* ^hypo/hypo^ mouse model. This increase in the metabolite/neurotransmitter ratios mainly results from the significant reduction in the levels of neurotransmitters without alteration in their corresponding metabolites (Supplementary Figure S4).

### 3.9 An Association Between the Hyperactive Behavior, Brain Neurotransmitters and Pls Levels in *Pex7* Deficient Mice

We repeated the open field test to evaluate the baseline activity levels in a subgroup of adult *Pex7* deficient mice prior to brain neurotransmitter analysis. Our results confirmed the hyperactive behavior in the *Pex7* hypomorphic mouse series compared to littermate controls (Figure 10A). Following the neurotransmitter analysis, a statistical correlation analysis was made to investigate the relation between the neurotransmitter deficits in cerebral cortex and activity levels. We found that the hyperactivity phenotype in *Pex7* deficient mice is inversely correlated with DA, NE, 5-HT, and GABA levels in cortical brain tissues (Figure 10C). Additionally, we observed a significant inverse correlation between hyperactivity and PlsEtn levels in plasma and cerebral cortex of *Pex7* deficient mice (Figure 10D).

### 3.10 Brain Neurotransmitter Deficits are Strongly Linked to Cerebral Cortex and Plasma Pls Levels in *Pex7* Deficient Mice

**FIGURE 11:**
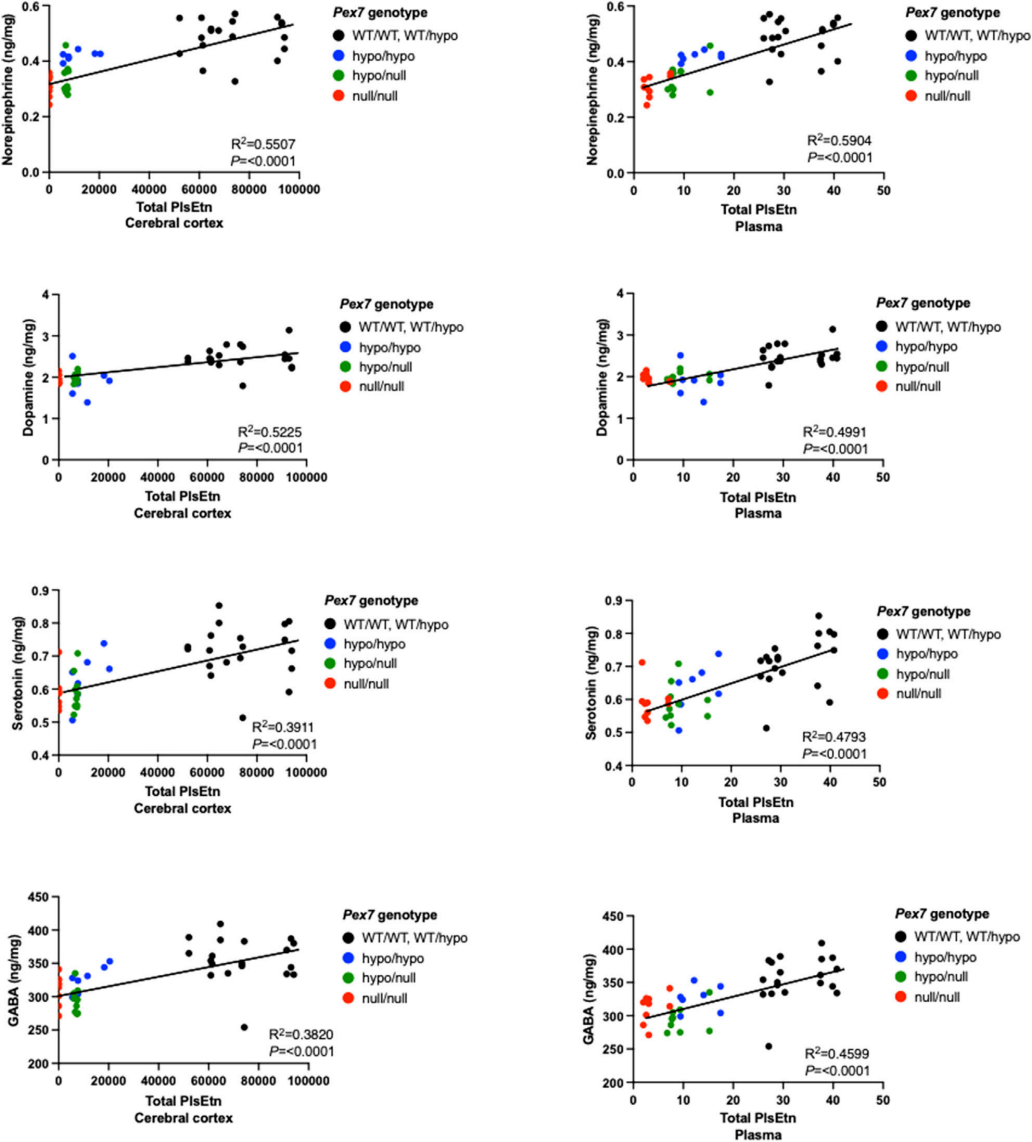
Brain neurotransmitter deficits correlate with total Pls levels in plasma and cerebral cortex samples from *Pex7* deficient mice. A significant positive correlation was found between the brain neurotransmitter levels and total PlsEtn measurements in plasma and cerebral cortex tissue from *Pex7* deficient mice. Total PlsEtn in cerebral cortex was measured as (nmol/mg protein) and in plasma was measured as (pmol). ****p < 0.0001 (*n* = 8–10 per genotype).

We further investigated the interrelation between brain neurotransmitter measurements and total PlsEtn levels in the cerebral cortex and plasma samples from *Pex7* deficient mice. We found that brain neurotransmitters deficits are positively and significantly correlated with the degree of Pls deficiency in cerebral cortex and plasma from *Pex7* deficient mice (Figure 11).

## 4 Discussion

We generated and evaluated an allelic series of *Pex7* hypomorphic mice to reflect the spectrum of human RCDP1 phenotypes. A comparative analysis of behavioral, biochemical, histological profiles of the nervous system was performed to highlight the effect of Pls deficiency particularly in the milder form of RCDP1. Furthermore, insight into disease pathophysiology can help to improve RCDP1 patient care.

### 4.1 Small Increases in *Pex7* Transcript and Protein Levels can Dramatically Improve Phenotypes

Gene expression studies of *Pex7* ^hypo/hypo^ and *Pex7* ^hypol/null^ revealed extremely low levels (<1%) of *Pex7* transcript in all tissues evaluated and undetectable *Pex7* transcript in *Pex7* ^null/null^. However, the high cycle quantification (Cq) values associated with *Pex7* relative expression (above 30) in the *Pex7* hypomorphic models, suggests that *Pex7* transcript levels were extremely low and could be beyond the limit of detection in qPCR analysis (Taylor et al., 2010). Consistent with the reduced transcript levels observed, PEX7 protein levels were very low in tissues from the *Pex7* ^hypo/hypo^ mice, and undetectable by immunoblotting in either *Pex7* ^hypo/null^ or *Pex7* ^null/null^. Although *Pex7* hypomorphic mice exhibited very low *Pex7* transcript and protein levels, the phenotypic manifestations were dramatically improved in comparison to *Pex7* ^null/null^. Overall, these data suggest that small increases in *Pex7* transcript and/or protein can markedly improve the clinical phenotype, which might be substantially beneficial to accelerate future therapeutic interventions.

### 4.2 Correlations Between *Pex7* Genotype Severity and Phenotypes

#### 4.2.1 Survival

The Pex*7* ^null/null^ had the highest mortality rate of 77% within the first 3 weeks of life, similar to the previously reported *Pex7* ^null/null^ model (Brites et al., 2003). The mortality rate in human patients with classic RCDP was 55% at the age 12 years (Duker et al., 2019). In individuals with mild (nonclassic) RCDP, the estimated mortality rate was only 9% by the end of childhood stage (Duker et al., 2019), similar to the 10% mortality rate that was observed in *Pex7* hypomorphic mice.

#### 4.2.2 Pls Levels

The concept that Pls deficiency plays a key role in determining the degree of phenotype severity in RCDP was first introduced in the initial genotype-phenotype correlation of human RCDP1 patients (Braverman et al., 2002). Accordingly, in previously reported RCDP mouse models, Pls levels were undetectable in *Gnpat* ^null/null^ and severely reduced levels to 5% in erythrocyte and 0.05% in brain tissues from *Pex7* ^null/null^ (Brites et al., 2003; Rodemer et al., 2003). We observed graded reduction of Pls levels in *Pex7* deficient mice according to the severity of RCDP.

#### 4.2.3 C26:0-LPC Levels as a Reflection of VLCFA Levels

Human RCDP1 patients typically do not show elevated VLCFA levels in plasma or have a measurable impairment in peroxisomal fatty acid β-oxidation in cultured skin fibroblasts. However in one study of 20 RCDP patients, a 2-fold increase in C26:0-LPC over controls in blood cells was observed, with normal levels in plasma and fibroblasts (Schutgens et al., 1993). We reported a transient elevation in C26:0-LPC with 1.5-fold increase over controls in erythrocytes from 4 mild RCDP patients (Fallatah et al., 2020). Transient VLCFA elevation was also reported in liver and brain tissues from *Pex7* null mice at birth, but normalized by age 2 months (Brites et al., 2003). In the current study, we showed that C26:0-LPC levels were elevated in accordance to genotype severity in *Pex7* deficient mice at 4 months of age. The extent of C26:0 elevation was variable between examined tissues and showed a range of 2- to 11-fold increase over WT in sampled peripheral tissues. Surprisingly, no accumulation of C26:0-LPC was found in cerebral cortex. We compared C26:0-LPC levels in our *Pex7* deficient mice to a previously reported mouse model of the peroxisome biogenesis disorder Zellweger Spectrum disorder (*Pex5* KO) and the peroxisomal enzyme/transporter deficiency X-linked adrenoleukodystrophy (*Abcd1* KO). *Pex5* KO mice showed a range of 3- to 9-fold increase over WT in C26:0 VLCFA in plasma and brain tissues at 3 months of age (Baes et al., 1997; Baes et al., 2002). *Abcd1* KO mice displayed 4- to 5-fold C26:0 increase over WT in brain, spinal cord, liver, spleen, kidney and testis at 6 months of age (Brites et al., 2009). Thus, the elevation of VLCFA in *Pex7* deficiency is distinguished by being absent in the cerebral cortex making it unlikely to contribute to the cortical phenotype in *Pex7* deficient mice. Tissue elevation is most likely due to the absence of ACAA1 thiolase without full compensation from SCPx, which is imported by PEX5. Tissue specific differences in VLCFA levels in the *Pex7* deficient mice could reflect tissue specific availability of the SCPx protein.

#### 4.2.4 PA Levels

To determine the significance of elevated PA, we compared levels in plasma and brain tissues from our *Pex7* deficient mice and previously reported ARD mouse model (*Phyh* ^null/null^ mice) with isolated defects in PA oxidation. To make the comparison of PA levels between *Pex7* deficient mice and ARD mice, we used fold change over WT which would adjust and correct for unit differences and make the comparison between studies possible. Seven-week-old ARD mice on standard chow showed an 80-fold increase over control in plasma with undetectable levels in cerebellar tissues (Ferdinandusse et al., 2008). We have shown that *Pex7* ^null/null^ have a gradual and significant accumulation of PA, with 6-fold increase at 1 month and 18-fold increase at 12 months of age compared to *Pex7* ^WT/WT^ in plasma, and 12- to 67-fold increase compared to *Pex7* ^WT/WT^ in brain. In plasma and brain tissues from *Pex7* ^hypo/null^ mice, there was a 3- and 5-fold increase respectively over *Pex7* ^WT/WT^ at 12 months of age. Thus, plasma PA levels accumulate in Pex7 deficiency and partially correlate to genotype but are higher in the ARD mouse model.

We found a small amount of PA (0.373 mg/100 g) in standard mouse chow. The total free and esterified phytol in standard mouse chow was 1.87 mg/100 g weight of mouse chow. If a mouse eats 4 g of chow per day, then they are getting around 80 μg of phytol/esters plus PA, equal to 96 μg per day. We concluded that even these small amounts of PA in rodent chow could lead to PA toxicity over time in *Pex7* deficient mice. The 6-fold–67-fold elevation over wild type (that is dependent on age and tissue) observed in our mice corresponds to the 20–100-fold elevation that can be seen in ARD patients on regular diets (Baldwin et al., 2010). The tolerable daily intake for ARD patients is less than 10 mg of phytol per day (Krauß et al., 2017). Also, PA levels in liver and brain tissue from 8-week-old *Pex1* hypomorphic mice on standard mouse chow were 100-fold and 7-fold elevated respectively compared to wild type mice (Moser et al. unpublished data). Thus, it is apparent that significant amounts of phytol are in standard mouse chow, and depending on the mice ability to degrade this, can accumulate in tissues.

The pathological consequences of elevated PA were investigated in ARD mice supplemented with 0.1%–0.25% phytol diet for 3–6 weeks (Ferdinandusse et al., 2008). Phytol-treated ARD mice exhibited unsteady gait with reduced base of support for the hind paws in the CatWalk system (ataxia), delayed motor nerve conduction velocity (peripheral neuropathy) and loss of cerebellar Purkinje cells. The authors of the study (Ferdinandusse et al., 2008) suggested that Purkinje cells loss could be a result of elevated PA and lead to the ataxia. We found that *Pex7* ^null/null^ mice displayed significant Purkinje cells loss by 4 months of age which was more prominent and associated with ataxia at age 12 months, as well as a significant elevation of PA. *Pex7* ^hypo/hypo^ and *Pex7* ^hypo/null^ mice had lower PA levels in cerebellum, a milder reduction in the number of cerebellar Purkinje cells and no gait abnormalities at age of 12 months. These data also suggest that PA accumulation to critical levels could result in Purkinje cells loss and clinical manifestation of cerebellar ataxia. In the ARD mouse model Purkinje cell loss occurred only after phytol supplementation and without the effect of Pls deficiency. In our *Pex7* deficient series, which did not receive phytol supplementation but did receive small amounts from standard chow, we proposed that Pls deficiency might exacerbate the effect of PA accumulation and subsequently lead to Purkinje cell loss as well as ataxic gait. Similarly, a previous report showed that Pls deficiency can aggravate the pathology of elevated VLCFA (Brites et al., 2009). We think that the comparison of PA levels between *Pex7* deficient mice and the ARD mouse model is a crucial step for clinical significance as it provides insight into the threshold at which PA levels could lead to neuropathological manifestation of Purkinje cell loss and clinical presentation of ataxia. PA levels could be a valuable clinical tool to manage in order to prevent the clinical manifestation of ataxia and Purkinje cell loss overtime particularly in patients with milder form of PEX7 deficiency.

Purkinje cell loss was previously reported in postmortem examinations of 3 patients with classic (severe) RCDP and it was suggested to be the causative factor of cerebellar atrophy found in RCDP patients although the levels of PA in 2 cases were not provided and mentioned to be high only in a single patient (Powers and Moser, 1998; Powers et al., 1999). It is possible that the development of cerebellar atrophy could be preventable by applying a PA-restricted diet.

### 4.3 Behavioral Abnormalities and Neurotransmitter Defects are Linked to Pls Deficiency

Neurobehavioral disorders including autism spectrum disorder (ASD) and attention deficit hyperactivity disorder (ADHD) were reported in patients with milder forms of RCDP (Bams-Mengerink et al., 2013; Yu et al., 2013). In addition, we found that 56% of mild RCDP individuals have behavioral abnormalities (Fallatah et al., 2020). Our results revealed that *Pex7* deficient mice displayed a hyperactive behavior, which also matches the previous report of a hyperactivity phenotype in the *Gnpat* ^null/null^ (RCDP2) mouse model (Dorninger et al., 2019b). Pls deficiency could be a contributing factor in the development of this hyperactive behavior through the role of PLs in regulating brain neurotransmitter homeostasis.

In this study we detected significant deficiencies in major brain neurotransmitters including DA, 5-HT, NE, and GABA in brain homogenates from *Pex7* deficient mice with a significant correlation to activity levels. Also, we found that deficits in NE and 5-HT were strongly correlated with Pls levels and thus the severity of Pex7 genotype. Similar neurotransmitter deficits were reported in *Gnpat* ^null/null^ mice and were proposed to be caused by defects in presynaptic neurotransmitter release and an impairment in vesicular uptake (Dorninger et al., 2019b). Brodde et al., (2012) showed an impairment in presynaptic release of glutamate and acetylcholine neurotransmitters in murine nerve terminals (synaptosomes) isolated from *Gnpat* ^null/null^ mice with reduction in exocytosis of endogenous acetylcholine and glutamate by 50%—60% of normal control synaptosomes. Additionally, *in vitro* radiolabeled neurotransmitter release studies of slices from hippocampus and parietal cortex of *Gnpat* ^null/null^ mice showed 22%–30% reduction in [^3^H] norepinephrine release upon chemical and electrical stimuli. Decreased levels of vesicular monoamine transporter type 2 (VMAT2) were observed in striatum from *Gnpat* ^null/null^ mice. VMAT2 is responsible for transporting monoamines from the cytosol to presynaptic vesicles and its reduction could affect the transport of neurotransmitters into synaptic vesicles therefore causing the depletion in monoamine neurotransmitters in Pls deficient mice (Dorninger et al., 2019b). Additionally, the hyperactive phenotype was previously reported in non-peroxisomal mouse models with deficiency in brain neurotransmitters including dopamine (Giros et al., 1996), 5-HT (Whitney et al., 2016) and GABA (Chen et al., 2015), which further supports the hypothesis of a direct role of brain neurotransmitter deficits in the manifestation of hyperactivity. For the future, it is possible to investigate RCDP patients for neurotransmitter deficits - urine, plasma or CSF can be analyzed for 5-HT, DA, NE as well as neurotransmitter metabolites (5-HIAA, HVA).

In the nervous system, Pls are considered as a critical source of the polyunsaturated omega-3 essential fatty acid DHA (22:6 n−3) that plays a crucial role in brain development, myelination and function (Hishikawa et al., 2017). In a prior cross-sectional study of 53 school age children with ADHD and age-matched normal controls, a lower proportion of plasma levels of DHA 22:6 *n*−3 was found in ADHD group (Burgess et al., 2000). Additionally, a deficiency of DHA in rhesus monkeys was associated with a stereotyped behavior (Reisbick et al., 1994). Also, previous studies indicated a deficiency of DHA in brain tissues and erythrocytes from *Gnpat* ^null/null^ (Rodemer et al., 2003) and hypomorphic *Pex7* mice (Braverman et al., 2010). In the current project, we show a reduction in all Pls in addition to DHA-containing Pls in brain tissues from *Pex7* deficient mice using LC-MS/MS. We propose that DHA deficiency might contribute to the development of the hyperactivity phenotype observed in *Pex7* deficient mice.

To summarize, we engineered an allelic series of Pex*7* deficient mice based on reductions of a normal Pex7 transcript, that mimics the human RCDP1 spectrum. *Pex7* deficient mice showed genotype-phenotype correlations in terms of survival, body weight, peroxisome metabolites (Pls, C26:0-LPC, and PA) neurochemical, neuroanatomical and neurobehavioral (neurotransmitter levels, myelination, Purkinje cells numbers, and activity levels) manifestations. This graded genotype-phenotype correlation provided a type of “dose response” that strengthened the association of the phenotypes with *Pex7* deficiency. The marked improvement in survival and body weight with only marginal changes in *Pex7* transcript and PEX7 protein levels from the null to the hypomorphic model should facilitate the efficacy of any future molecular therapies in RCDP.

Our results of the hyperactivity behavior in *Pex7* deficient mice suggest that reduced Pls levels play a significant role in the development of behavioral dysfunction associated with RCDP. This hyperactivity is sensitive to small reductions in Pls levels and could represent the neurobehavioral phenotype observed in mild (nonclassic) RCDP patients. We recently showed that oral supplementation of a mature synthetic Pls improved Pls levels in plasma, erythrocyte, heart, liver, small intestine and skeletal muscle tissues from *Pex7* ^hypo/null^ mice (Fallatah et al., 2019). Although we did not observe an augmentation in Pls levels in the cerebral cortex and cerebellum, mature Pls effectively normalized the hyperactive behavioral phenotype in *Pex7* deficient mice.

Our data highlight the significant correlation that was observed in *Pex7* deficient mice between the hyperactivity, brain neurotransmitter deficits and Pls deficiency. These key findings emphasize the mechanistic role of Pls deficiency in regulating brain function and behavior. While the underlying mechanisms of PEX7 defects as well as Pls deficiency in complex brain behavior is still unknown, we speculate that Pls deficiency results in altered membrane lipid composition that could directly influence the basic physiological function of cellular membranes in the nervous system. Additionally, Pls deficiency might affect the overall cellular signaling as well as intracellular pathways and synaptic release and transmission, which might lead to impairments in brain function.

## Data Availability

The original contributions presented in the study are included in the article/Supplementary Material, further inquiries can be directed to the corresponding authors.
