# Supplementary material for: A Pex7 Deficient Mouse Series Correlates Biochemical and Neurobehavioral Markers to Genotype Severity—Implications for the Disease Spectrum of Rhizomelic Chondrodysplasia Punctata Type 1
Source: Front Cell Dev Biol. 2022 Jul 11;10:886316. doi: 10.3389/fcell.2022.886316 (PMC9310236; doi:10.3389/fcell.2022.886316)
Supplement: Supplementary file 1 [file DataSheet1.docx]

**Supplementary data**

**Supplementary Table S1. List of primers used in this study.**

| **Primers combinations for PCR *Pex7* genotyping^*^** | |
| --- | --- |
| **4270F** | 5′- CAGACTGCCTTG GGAAAAG -3′ |
| **7435R** | 5′- GTGTCCATCACTCAACCTGG -3′ |
| **7902F** | 5′- GCTTTGACTGGAATGACGGC -3′ |
| **5269R** | 5′- GCACTGCTGTTGCCTAATGA -3′ |
| **2033F** | 5′- GGGGCAAGTCTTTTGAAGTC -3′ |
| **2561R** | 5′- GAGCAGGTAGTGGCTTTCTA -3′ |
| **2071NeoF** | 5′- GGCACACTTCTTCCAGCAATA -3′ |
| **2811NeoR** | 5′- GGTGGGGTGGGATTAGATAAA -3′ |
| **Primers for quantitative real-time PCR** | |
| ***Pex7*F** (Exon 1-2) | 5′- CATCGCGGGCTGTGGAAC -3′ |
| ***Pex7*R** (Exon 4) | 5′- ACAAGCCGTCATTCCAGTCAA -3′ |
| ***Hprt*F** | 5′- TGCTGACCTGCTGGATTACA -3′ |
| ***Hprt*R** | 5′- TTATGTCCCCCGTTGACTGA -3′ |

^*^The forward and reverse primers listed in the table were paired into the following different combinations to determine the genotype of *Pex7* deficient mice and their littermate controls: P1 (4270F×5269R) was used to screen mice for null allele (with deleted exon 3), P2 (7902F×5269R) would amplify the allele segment containing the exon 3 to screen mice without the null allele, P3 (4270F×7435R) would specifically amplify the *Pex7* allele containing both the neomycin cassette and the proximal loxP amplicon, to screen for the hypomorphic allele. P4 (2033F×2561R) would amplify the *Pex7* wild-type allele, and P5 (2071NeoF×2811NeoR) would amplify the neomycin cassette and the proximal loxP amplicon.

**Supplementary Figure S1.** **Tissue-specific distribution of total phosphatidylethanolamine plasmalogens (PlsEtn) species in *Pex7* deficient mice.** Total PlsEtn levels in several tissues from *Pex7* deficient mice correlate with the severity of the genotype. Significant reduction in PlsEtn levels was observed in *Pex7* deficient mice compared to their littermate controls (P < 0.0001) (n=4-6 per genotype). Bars represent group means ± SD. C16:0, C18:0 and C18:1 represent the sum of individual measurements of the PlsEtn species with C16:0, C18:0 or C18:1 fatty alcohol at the sn-1 position. The C22:6 represents the sum of the PlsEtn measured with C22:6 fatty acid side chains at the sn-2 position.


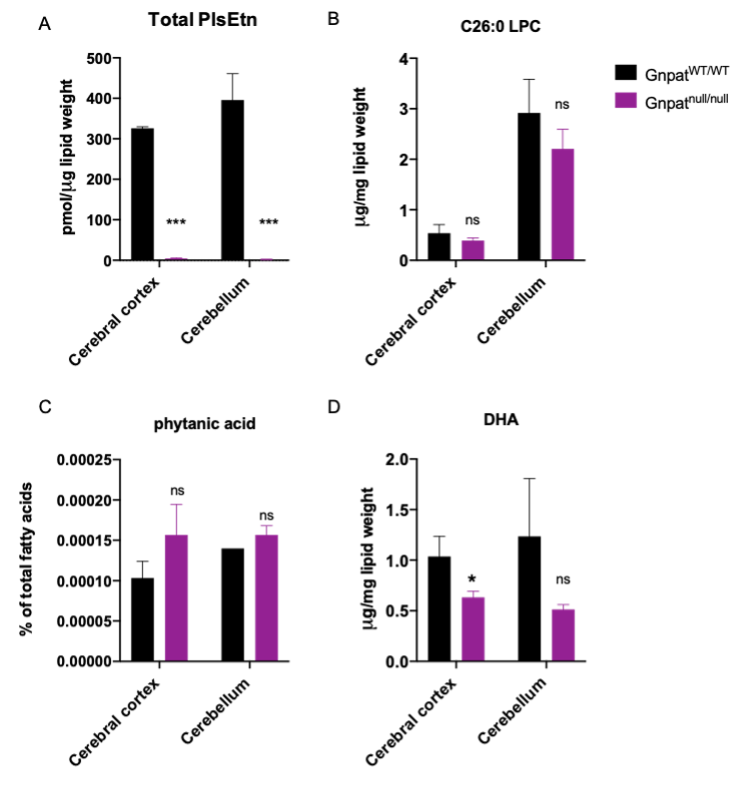


**Supplementary Figure S2. Peroxisome metabolites in the brain tissues of *Gnpat*^null/null^ mice.** *Gnpat*^null /null^ mice showed a significant deficiency in Pls and DHA levels. No accumulation of either C26:0-LPC or PA levels was found in the brain tissues from *Gnpat*^null/null^ mice compared to their littermate wild type *Gnpat*^WT/WT^ mice. ns: nonsignificant, **P* < 0.05, ***P* < 0.01, ****P* < 0.001 (n=3 per genotype, age: 4 months).

A


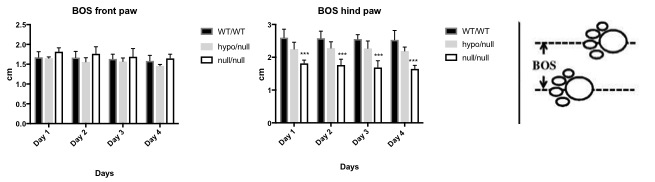


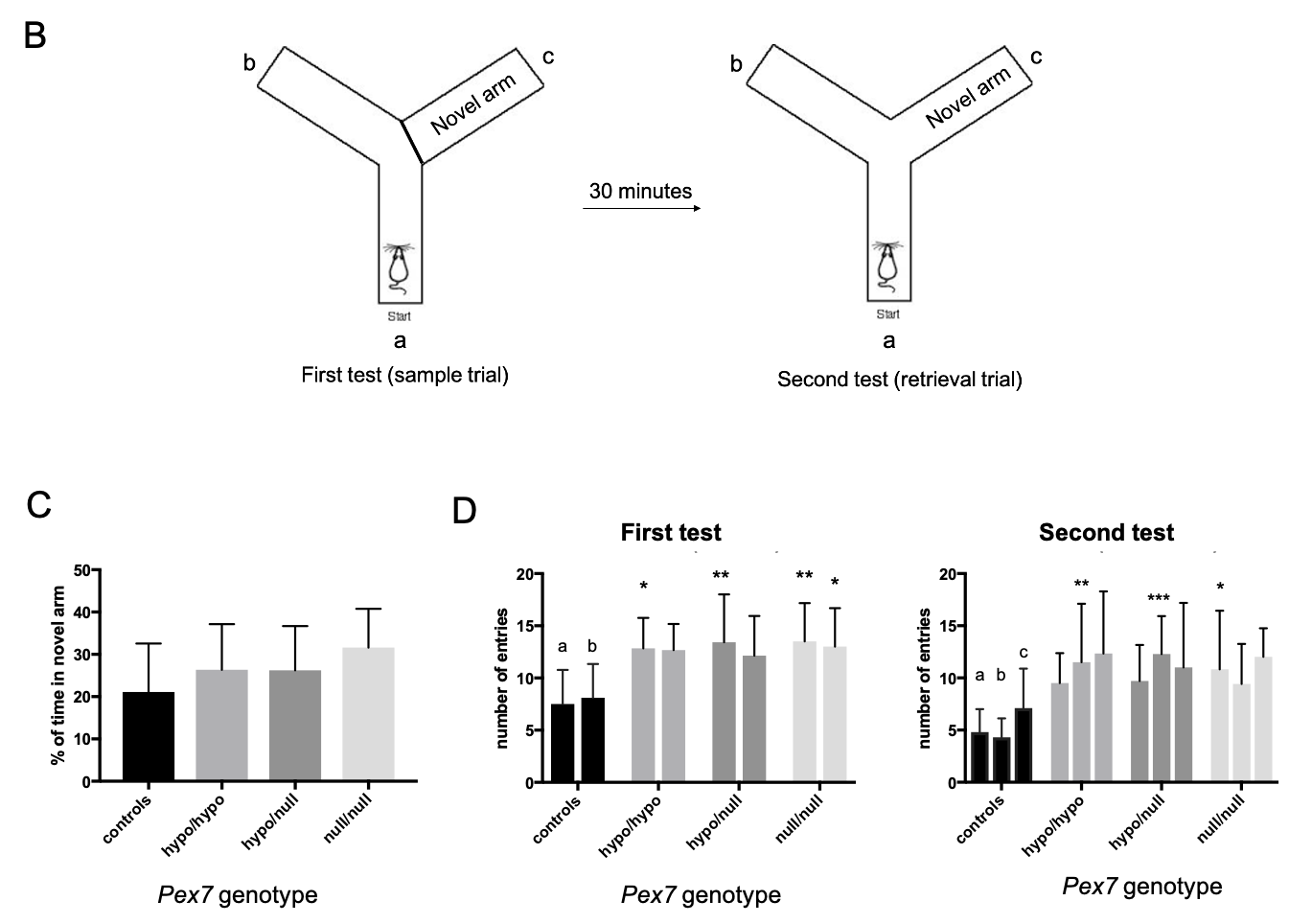


**Supplementary Figure S3. *Pex7* deficient mice showed normal memory function in Y-maze test and *Pex7*^null/null^ displayed ataxic gait in the CatWalk gait analysis. A)** Base of support (BOS) for the hind paws was significantly decreased in the severe (*Pex7*^null/null^), but not the hypomorphic (*Pex7*^hypo/null^) mouse model at 12 months of age compared with the BOS of *Pex7*^WT/WT^. There were no significant differences for BOS of the front paws. **B)** Schematic representation of the Y-maze forced alternation test. **C)** No significant differences were observed in the percentage of time in the novel arm. **D)** Increased number of entries was found in *Pex7* deficient mice compared to littermate controls (*Pex7*^WT/WT and^ *Pex7*^WT/hypo^). **a** represents the entry arm, **b** represents the additional arm and **c** represents the novel arm of the Y-maze test. A statistical comparison was made between the number of entries in each arm in *Pex7* deficient mice compared to the number of entries of each arm in *Pex7* controls. The percentage of time in the novel arm was calculated as the time spent in the novel arm divided by the time spent in all arms during the second test. **P* < 0.05, ***P* < 0.01, ****P* < 0.001, (n=8-10 per genotype).


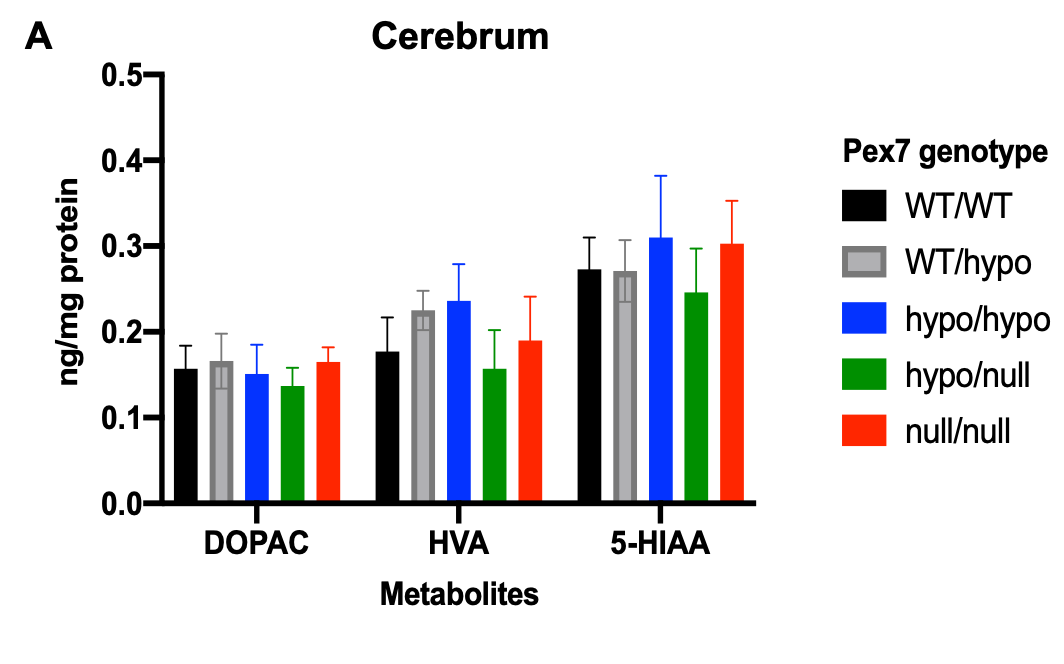


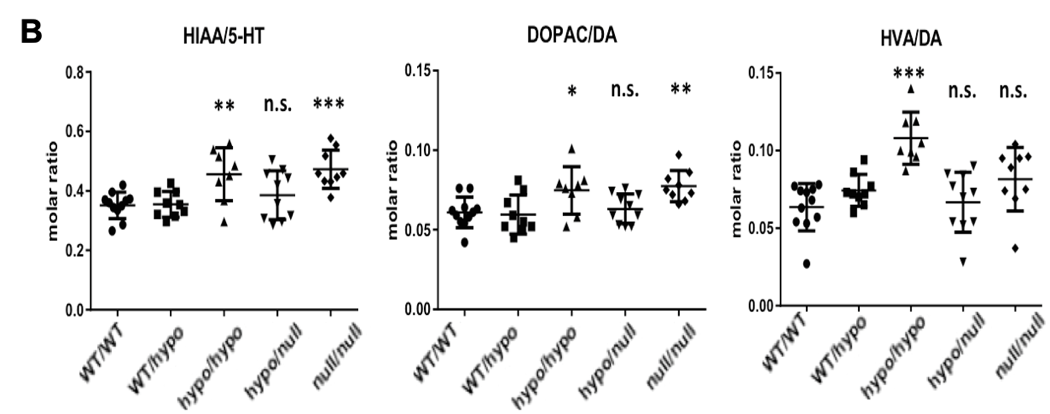


**Supplementary Figure S4. Increased turnover of monoamine brain neurotransmitters in *Pex7* deficient mice.** **A)** No significant differences in the baseline levels of metabolites that correspond to brain monoamines neurotransmitters in whole brain tissue homogenates from *Pex7* deficient mice compared to their littermate *Pex7* controls (*Pex7*^WT/WT and^ *Pex7*^WT/hypo^). **B)** Ratio of monoamine metabolites to their respective transmitters. This ratio reflects the increase in turnover of monoamine neurotransmitters in *Pex7* deficient mice. 5-HIAA: 5-hydroxyindoleacetic acid, the main metabolite of serotonin (5-HT), DOPAC: 3,4-dihydroxyphenylacetic acid and HVA: homovanillic acid, the primary metabolites for dopamine (DA). **P* < 0.05, ***P* < 0.01, ****P* < 0.001, (n=8-10 per genotype).
